# The phosphorelay BarA/SirA activates the non-cognate regulator RcsB in *Salmonella enterica*

**DOI:** 10.1371/journal.pgen.1008722

**Published:** 2020-05-11

**Authors:** Hubert Salvail, Eduardo A. Groisman

**Affiliations:** 1 Department of Microbial Pathogenesis, Yale School of Medicine, New Haven, Connecticut, United States of America; 2 Yale Microbial Sciences Institute, West Haven, Connecticut, United States of America; Michigan State University, UNITED STATES

## Abstract

To survive an environmental stress, organisms must detect the stress and mount an appropriate response. One way that bacteria do so is by phosphorelay systems that respond to a stress by activating a regulator that modifies gene expression. To ensure an appropriate response, a given regulator is typically activated solely by its cognate phosphorelay protein(s). However, we now report that the regulator RcsB is activated by both cognate and non-cognate phosphorelay proteins, depending on the condition experienced by the bacterium *Salmonella enterica* serovar Typhimurium. The RcsC and RcsD proteins form a phosphorelay that activates their cognate regulator RcsB in response to outer membrane stress and cell wall perturbations, conditions *Salmonella* experiences during infection. Surprisingly, the non-cognate phosphorelay protein BarA activates RcsB during logarithmic growth in Luria-Bertani medium in three ways. That is, BarA’s cognate regulator SirA promotes transcription of the *rcsDB* operon; the SirA-dependent regulatory RNAs CsrB and CsrC further increase RcsB-activated gene transcription; and BarA activates RcsB independently of the RcsC, RcsD, and SirA proteins. Activation of a regulator by multiple sensors broadens the spectrum of environments in which a set of genes is expressed without evolving binding sites for different regulators at each of these genes.

## Author summary

The phosphorelay is a form of signal transduction used by organisms in all three domains of life. Typically, a phosphorelay consists of sensor proteins that respond to specific signals by activating a cognate regulatory protein that alters gene expression. Phosphorelays exhibit specificity towards their cognate regulators, thereby ensuring that any changes in gene expression help an organism cope with the experienced stress (and not with an unrelated stress). However, we now report that the regulator RcsB is activated by both cognate and non-cognate phosphorelay proteins in the bacterium *Salmonella enterica* serovar Typhimurium. The phosphorelay proteins RcsC and RcsD activate RcsB upon cell envelope perturbations, whereas the non-cognate phosphorelay protein BarA activates RcsB during rapid growth in Luria-Bertani medium. Our findings establish that BarA controls gene expression via both its cognate regulator SirA and the non-cognate regulator RcsB. In addition, they demonstrate that RcsB controls gene expression in response to multiple signals detected by the RcsC, RcsD, and BarA proteins.

## Introduction

Survival in a hostile environment requires gene products that protect an organism from the particular stresses present in that environment. Phosphorelay systems allow bacteria to respond to environmental insults by activating a regulatory protein that alters the expression of a subset of genes [1, 2]. To ensure a specific response to a given stress condition, phosphorelay systems usually restrict activation to their respective cognate regulatory proteins [3–5]. However, we now report that a regulatory protein is activated both by cognate and non-cognate phosphorelay proteins depending on the stress experienced by the bacterium *Salmonella enterica* serovar Typhimurium.

Bacteria and cell wall-containing eukaryotes utilize two-component systems to alter behavior in response to changes in environmental or cellular conditions [6, 7]. The prototypical two-component system consists of a sensor that responds to a specific signal(s) by modifying the activity of a cognate regulator through phosphorylation. Phosphorelays are versions of two-component systems in which there is a three-step phosphotransfer, whereby the phosphoryl group is shuttled from the sensor kinase to the response regulator via an intermediary phosphotransferase protein or domain, as opposed to the single phosphotransfer event from a sensor to a regulator in classical two-component systems [8, 9].

**Fig 1:**
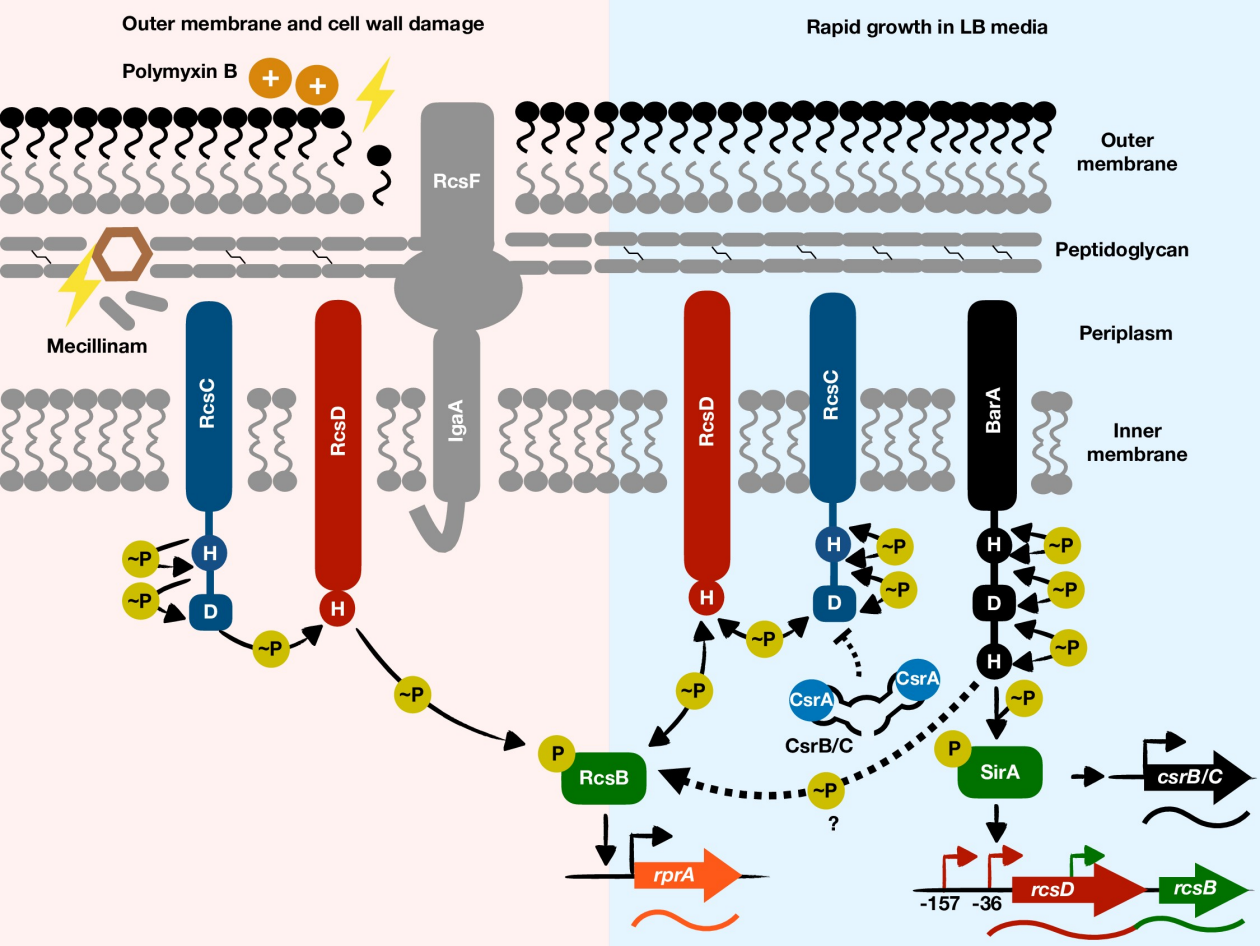
The phosphorelay BarA/SirA activates the non-cognate regulator RcsB. Upon polymyxin B-induced outer membrane damage or mecillinam-induced cell wall damage (left panel), the sensor kinase RcsC and the phosphotransferase RcsD activate their cognate response regulator RcsB, which then alters transcription of its target genes (e.g., *rprA*). The single-headed arrows for the RcsC/RcsD/RcsB phosphotransfer cascade indicate that upon outer membrane and cell wall damage, the phosphate flow is directed toward RcsB. Under these conditions, BarA is dispensable for RcsB activation. Upon rapid growth in LB media (right panel), BarA uses multiple mechanisms to activate RcsB. That is, BarA’s cognate regulator SirA promotes transcription of the *rcsDB* operon, increasing the amounts of the RcsD and RcsB proteins; the SirA-dependent regulatory RNAs CsrB and CsrC reduce RcsC expression, probably through titration of the CsrA protein, thus limiting RcsB dephosphorylation by RcsC and RcsD. BarA also activates RcsB independently of the RcsC, RcsD, and SirA proteins. The latter mechanism may entail direct phosphotransfer from BarA to RcsB and is depicted by a dashed arrow and a question mark. The double-headed arrows for the RcsC/RcsD/RcsB phosphotransfer cascade indicate that upon rapid growth in LB media, the phosphate flow may be reversed, resulting in the phosphate being taken away from RcsB by RcsD and RcsC. RcsF is an outer membrane lipoprotein that senses envelope stress. IgaA is a negative regulator of the RcsC/RcsD/RcsB phosphorelay.

One of the best-studied phosphorelays in the family *Enterobacteriacae* is Rcs, which consists of the RcsC, RcsD and RcsB proteins [10, 11] (Fig 1). RcsC and RcsD are inner membrane proteins, whereas RcsB is a cytoplasmic DNA binding regulator that alters expression of its target genes when bacteria experience outer membrane stress or cell wall perturbations. These stresses are believed to promote RcsC autophosphorylation at a histidine residue followed by phosphotransfer to an aspartate residue within RcsC, followed by phosphotransfer to a histidine residue in RcsD, and ending in phosphotransfer to an aspartate residue on RcsB. Phosphorylated RcsB activates or represses a subset of genes involved in numerous cellular processes, including motility, biofilm formation, and the general stress response [3, 12–14]. Surprisingly, full expression of RcsB-activated genes has been observed in mutants lacking either the *rcsC* or *rcsD* gene [15, 16], raising the possibility of RcsB being activated by signaling proteins other than RcsC and RcsD.

We now report that the sensor phosphorelay protein BarA activates the non-cognate regulator RcsB in an RcsC- and RcsD-independent manner. We establish that BarA utilizes several mechanisms to increase the amounts of active RcsB protein. And we identify conditions in which RcsB activation requires RcsC and RcsD but not BarA. Our findings indicate that a given regulatory protein can be activated by non-cognate phosphorelay proteins. This activation expands the spectrum of environments in which a given set of genes is expressed without an organism needing to evolve binding sites for different regulatory proteins at the regulatory region of each target gene. Because the proteins that constitute a given phosphorelay are often encoded by independently transcribed genes, they may be more prone to establish physiological interactions with “non-cognate” partners.

## Results

### A condition that activates the regulator RcsB independently of the RcsC, RcsD, and RcsF proteins

We hypothesized that RcsB is activated by a non-cognate sensor because RcsB-dependent genes are fully expressed in the absence of either the *rcsC* or *rcsD* gene [15, 16], and also because the possibility of RcsC phosphorylating RcsB in the absence of RcsD, or of RcsD phosphorylating RcsB in the absence of RcsC [16], seemed unlikely [10].

To test this hypothesis, we examined the fluorescence of isogenic *Salmonella* strains with mutations in various *rcs* genes and harboring a medium copy number plasmid with a transcriptional fusion between the RcsB-activated *rprA* promoter and a promoterless *gfp* gene. Although the RcsB protein can form heterodimers with several proteins [10, 17, 18], *rprA* transcription is carried out by RcsB homodimers [15], which are favored upon RcsB phosphorylation [10]. Bacteria were grown in regular (i.e., NaCl-containing) Luria-Bertani (LB) media and in LB without NaCl as a means to decrease osmolarity.

**Fig 2:**
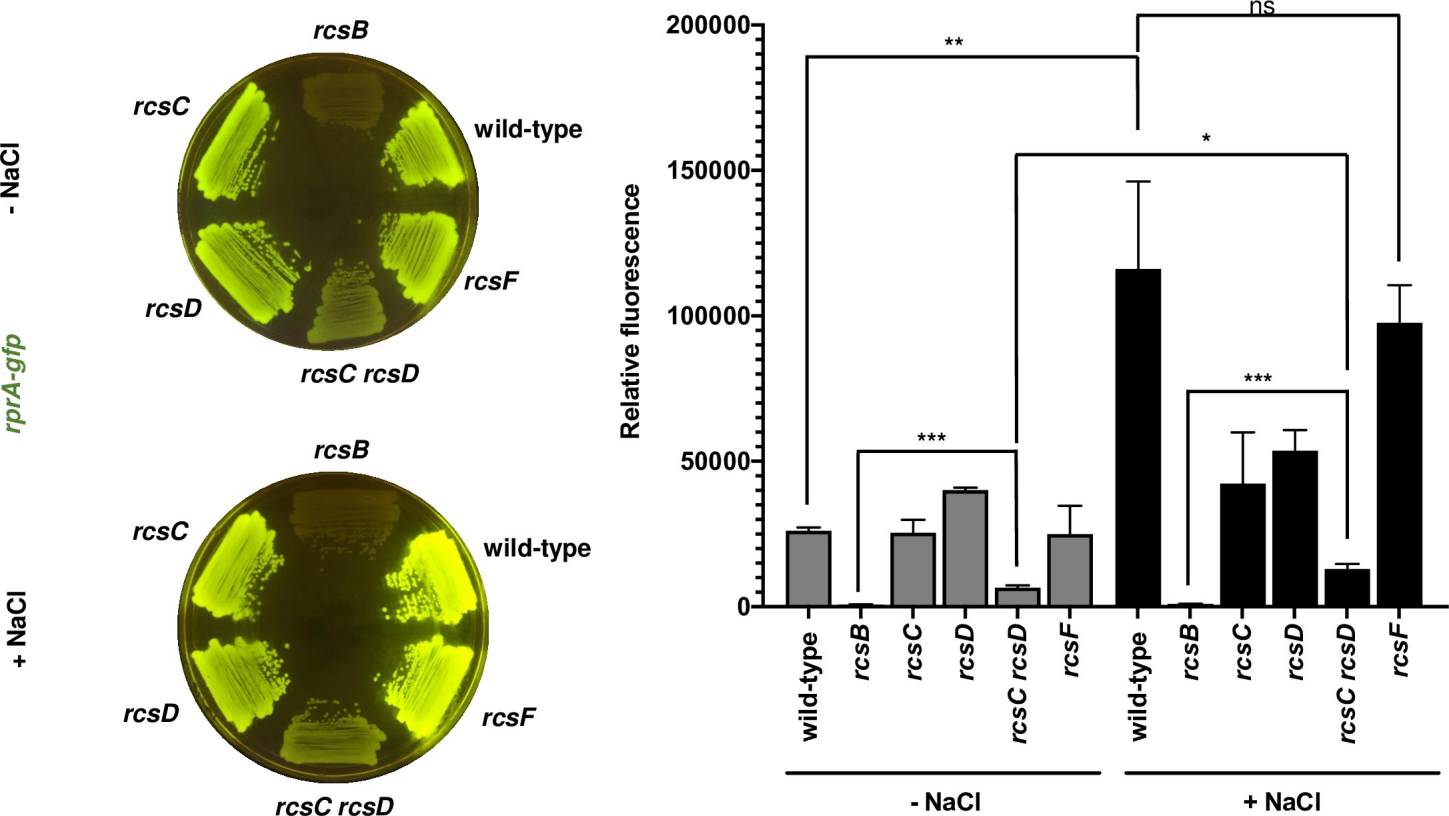
The response regulator RcsB can be activated independently of the RcsC, RcsD, and RcsF proteins. Fluorescence from wild-type (14028s), *rcsB* (EG12925), *rcsC* (HS1350), *rcsD* (HS1382), *rcsC rcsD* (HS1383) and *rcsF* (HS1326) *Salmonella* harboring plasmid pRprA-GFP (*rprA-gfp*) following 24 h of growth on LB solid medium without (-NaCl) or with (+NaCl) NaCl. Data are representative of three independent experiments, which gave similar results. Quantification of the fluorescence is provided on the right panel of the figure. Values derived from three independent experiments (mean ± standard deviation) were statistically analyzed by Prism 8 using two-tailed unpaired *t* test. Statistical significance is indicated by * *P*<0.05, **P<0.01, *** *P*<0.001; ns, not significant. Error bars indicate standard deviation.

Wild-type *Salmonella* fluoresced when incubated on LB agar plates lacking NaCl at 37˚C for 24 h (Fig 2). Fluorescence was 4-fold higher on regular (i.e., NaCl-containing) LB plates than in LB plates lacking NaCl. These results are in agreement with the notion that increased osmolarity activates the Rcs system [19, 20] (Fig 2). The *rcsB* mutant did not fluoresce on either plate (Fig 2), demonstrating that *rprA* transcription is fully dependent on RcsB under the two conditions [14, 15]. By contrast, the *rcsC* and *rcsD* single mutants exhibited wild-type fluorescence on NaCl-lacking LB plates (Fig 2). While displaying half of the wild-type strain fluorescence on LB plates, the fluorescence of the *rcsC* and *rcsD* single mutants was higher than that of the *rcsB* mutant (Fig 2). These results indicate that the RcsB-dependent *rprA* gene is still expressed in the absence of either RcsC or RcsD [15, 16].

The fluorescence of the *rcsC rcsD* double mutant was 2-fold higher on regular LB than in NaCl-lacking LB plates (Fig 2). Although this mutant had dramatically lower fluorescence than the wild-type strain or the *rcsC* and *rcsD* single mutants (Fig 2), its fluorescence was still higher than that of the *rcsB* mutant (Fig 2), reinforcing the notion that RcsB can be activated independently of RcsC and RcsD. These data argue that increased osmolarity activates RcsB by a non-canonical pathway.

The lipoprotein RcsF responds to envelope stress by activating the Rcs phosphorelay [11, 21, 22]. However, a *rcsF* null mutant retained wild-type fluorescence in both regular LB and NaCl-lacking LB plates (Fig 2). Taken together, these results demonstrate that RcsB can be activated by a pathway not involving the established players RcsC, RcsD, and RcsF.

### RcsB activation by the phosphorelay sensor BarA

To identify the gene(s) responsible for the RcsB-dependent RcsC- and RcsD-independent activation of the *rprA* promoter, we screened ~9,000 transposon Tn*10d*Tc-generated mutants of the *rcsC rcsD* strain carrying the *rprA-gfp* fusion for decreased fluorescence on LB agar plates. Mutants exhibiting less fluorescence than the parental strain were further characterized by transducing the transposon insertion into the original strain, and upon recapitulation of the phenotype, the site of insertion of the Tn*10d*Tc transposon was determined as described in Material and Methods.

One mutant had a total loss of fluorescence and, not surprisingly, harbored a Tn*10d*Tc insertion in the *rcsB* gene (S1 Fig). Another mutant, exhibiting a slight decrease in fluorescence, had a transposon insertion in *barA* (S1 Fig), encoding the tripartite sensor kinase BarA that forms a phosphorelay with the regulator SirA (known as UvrY in *E*. *coli*) [23, 24]. The BarA/SirA system has been implicated in motility, stress resistance, metabolism, and virulence [23, 25–27], some of which also require a functional RcsB protein [10, 28].

**Fig 3:**
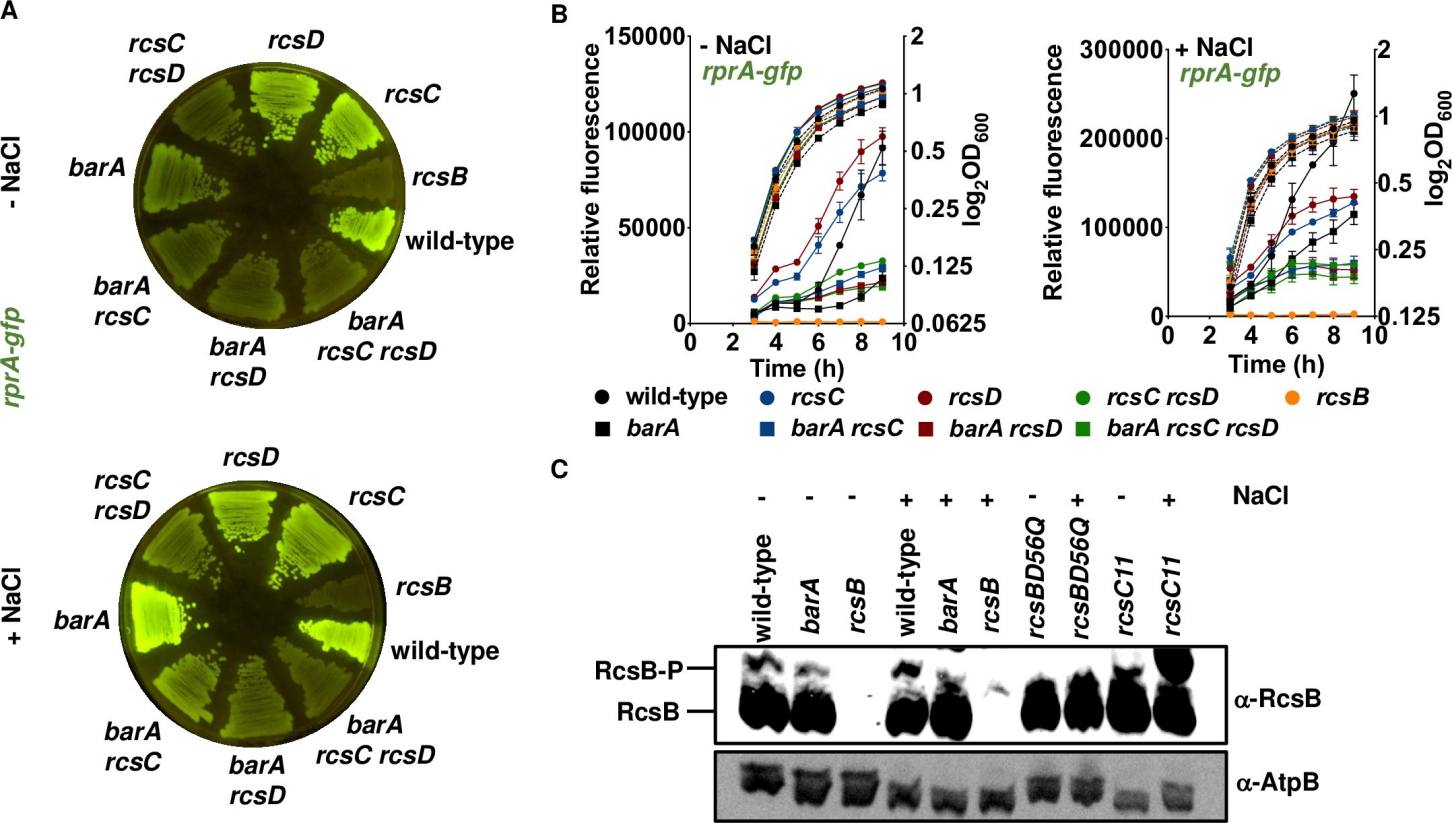
RcsB activation by the phosphorelay sensor BarA. (A) BarA is required for full RcsB activation on LB agar plates. Fluorescence from wild-type (14028s), *rcsB* (EG12925), *rcsC* (HS1350), *rcsD* (HS1382), *rcsC rcsD* (HS1383), *barA* (HS1520), *barA rcsC* (HS1521), *barA rcsD* (HS1522) and *barA rcsC rcsD* (HS1523) *Salmonella* harboring plasmid pRprA-GFP (*rprA-gfp*) following 24 h of growth on LB solid medium without (-NaCl) or with (+NaCl) NaCl. Data are representative of two independent experiments, which gave similar results. (B) BarA activates RcsB in a time-dependent manner. Fluorescence from wild-type (14028s), *rcsB* (EG12925), *rcsC* (HS1350), *rcsD* (HS1382), *rcsC rcsD* (HS1383), *barA* (HS1520), *barA rcsC* (HS1521), *barA rcsD* (HS1522) and *barA rcsC rcsD* (HS1523) *Salmonella* harboring plasmid pRprA-GFP (*rprA-gfp*) or pVector (empty pFPV25 vector) following 9 h of growth in LB liquid medium without (-NaCl) or with (+NaCl) NaCl. For each strain, relative fluorescence was obtained by subtracting the fluorescence of the strain harboring pVector from the fluorescence of the strain harboring pRprA-GFP. The obtained value was then divided by OD_600_. Error bars represent standard deviation from three independent experiments. Values derived from three independent experiments (mean ± standard deviation) were statistically analyzed by Prism 8 using two-tailed unpaired *t* test. Significance values (*P*) are reported in the text. Relative fluorescence values (left axis) are represented by solid lines and OD_600_ values (right axis) by dotted lines. (C) Western blot analysis of extracts prepared from wild-type (14028s), *barA* (HS1520) and *rcsB* (EG12925) grown in LB liquid medium without (-NaCl) or with (+NaCl) NaCl in late exponential phase (OD_600_ of ~1.0) following separation on Phos-tag SDS-PAGE to detect RcsB and RcsB-P. Samples were analyzed with antibodies directed to RcsB or AtpB proteins. Crude extracts from *rcsBD56Q* (HS1483) and *rcsC11* (EG14873) strains overnight cultures were used as negative and positive controls for RcsB phosphorylation, respectively. The *rcsBD56Q* mutant encodes an RcsB protein that cannot be phosphorylated at the conserved aspartate located at position 56 [14] and the *rcsC11* strain harbors the *rcsC11* point mutation promoting constitutive Rcs system activation [67].

When the *barA* gene was deleted from the *rcsC rcsD* double mutant, the resulting triple mutant exhibited less fluorescence than the *rcsC rcsD* double mutant on LB agar plates (Fig 3A), recapitulating the behavior of the *rcsC rcsD barA*::Tn*10d*Tc triple mutant (S1 Fig). These results indicate that the phenotype of the *rcsC rcsD barA*::Tn*10d*Tc triple mutant is due to loss of *barA* function (rather than to the *barA*::Tn*10d*Tc strain specifying a truncated BarA protein with altered activity).

The *barA* deletion mutant in an otherwise wild-type background had lower fluorescence than wild-type *Salmonella* on NaCl-lacking LB plates (Fig 3A) but similar fluorescence on regular LB plates (Fig 3A). The phenotype of the *barA* mutant is due to the absence of the *barA* gene because a plasmid with a copy of the wild-type *barA* coding region under the control of the *cat* promoter (pBarA) complemented the mutant, whereas the vector control (pVector) did not (S2 Fig). Fluorescence was also higher in wild-type *Salmonella* carrying pBarA than in the pVector control strain (S2 Fig). Given that the absence of *barA* decreases RcsB-dependent gene transcription in the presence of functional RcsC and RcsD proteins, BarA is a physiological activator of RcsB.

The *barA rcsC* and *barA rcsD* double mutants displayed lower fluorescence than the isogenic *rcsC* and *rcsD* single mutants, respectively, on both LB and NaCl-lacking LB plates (Fig 3A, S8 Fig and S12 Fig). By contrast, the *barA rcsC rcsD* triple mutant exhibited less fluorescence than the *rcsC rcsD* double mutant strain in LB but not in NaCl-lacking LB plates (Fig 3A).

As a member of the response regulator family, RcsB harbors a conserved aspartate residue that is phosphorylated when a bacterium experiences inducing conditions for its cognate phosphorelay proteins [10]. We have now determined that the BarA-mediated activation of RcsB requires the conserved aspartate in RcsB that is the site of phosphorylation. This is because fluorescence was much higher in wild-type *Salmonella* carrying the *rprA-gfp* fusion plasmid and pBarA than with the pVector control (S3 Fig), but similarly low in a *rcsBD56Q* genetic background (S3 Fig). The *rcsBD56Q* mutant encodes an RcsB protein that cannot be phosphorylated at the conserved aspartate located at position 56 [14] from the normal chromosomal location and promoter. Thus, RcsB’s phosphorylation site is necessary for activation by BarA.

We conclude that RcsB activation by BarA is condition specific, that LB solid media is an activating condition for BarA, that this activation is impacted by the presence of the phosphorelay proteins RcsC and RcsD, and that it requires the site of phosphorylation in RcsB.

### BarA activates RcsB in a time-dependent manner

To determine the kinetics of BarA activation of RcsB, we investigated the fluorescence of wild-type *Salmonella* carrying the *rprA-gfp* plasmid at different times after bacteria were inoculated into LB broth media with and without NaCl. Fluorescence was higher during growth in LB as compared to LB without NaCl (Fig 3B), in agreement with the results obtained on solid media (Fig 2). The *rcsB* mutant displayed no fluorescence over the 9 h course of the experiment (Fig 3B), as expected for the RcsB dependence of the *rprA* promoter (Fig 2) [14, 15].

In NaCl-lacking LB, the *rcsC* and *rcsD* single mutants showed 2- to 3-fold more fluorescence than the wild-type strain from 3 to 7 h (*P*<0.01 for *rcsC* versus wild-type at 3 h; *P*<0.001 for *rcsD* versus wild-type at 3 h; *P*<0.05 for *rcsC* versus wild-type at 7 h; *P*<0.01 for *rcsD* versus wild-type at 7 h) (Fig 3B). This result is consistent with the proposed reverse phosphate flow, whereby RcsD removes the phosphate from RcsB, and RcsC from RcsD [10, 15], which, intriguingly, is not observed on solid media (Fig 2). After 9 h, however, the *rcsC* and *rcsD* single mutants exhibited similar fluorescence as wild-type *Salmonella*, indicating that RcsB can be activated in the absence of either RcsC or RcsD (Fig 3B).

In contrast to the behavior manifested in NaCl-lacking LB, the *rcsC* and *rcsD* single mutants displayed wild-type fluorescence from 3 to 6 h in LB (Fig 3B). The fluorescence of these mutants was two-thirds to one-half that of the wild-type strain from 7 to 9 h (*P*<0.0001 for *rcsC* versus wild-type at 7 h; *P*<0.01 for *rcsD* versus wild-type at 7 h; *P*<0.001 for *rcsC* versus wild-type at 9 h; *P*<0.001 for *rcsD* versus wild-type at 9 h) (Fig 3B), arguing that both RcsC and RcsD are necessary for full RcsB activation in LB broth. These data also show that either RcsC or RcsD is sufficient to carry out RcsB activation during the first 6 h.

The *rcsC rcsD* double mutant exhibited the same fluorescence as the wild-type strain from 3 to 6 h in NaCl-lacking LB, demonstrating that RcsB can be activated independently of both RcsC and RcsD (Fig 3B). From 7 to 9 h, the fluorescence of the *rcsC rcsD* double mutant was two-thirds to one-third that of the *rcsC* and *rcsD* single mutants or of wild-type *Salmonella* (*P*<0.001 for *rcsC rcsD* versus *rcsC* at 7 h; *P*<0.0001 for *rcsC rcsD* versus *rcsD* at 7 h; *P*<0.01 for *rcsC rcsD* versus wild-type at 7 h; *P*<0.0001 for *rcsC rcsD* versus *rcsC* at 9 h; *P*<0.0001 for *rcsC rcsD* versus *rcsD* at 9 h; *P*<0.001 for *rcsC rcsD* versus wild-type at 9 h) (Fig 3B), implying that either RcsC or RcsD is sufficient to achieve full RcsB activation under these conditions. Although wild-type *Salmonella* and the *rcsC rcsD* double mutant exhibited a similar fluorescence at the beginning of growth in LB, fluorescence was 3- to 4-fold higher in wild-type *Salmonella* than in the double mutant from 6 to 9 h (*P*<0.01 for *rcsC rcsD* versus wild-type at 6 h; *P*<0.0001 for *rcsC rcsD* versus wild-type at 9 h) (Fig 3B).

The *barA* mutant strain exhibited lower fluorescence than wild-type *Salmonella* both in LB *(barA*/wild-type ratio of 0.5 at 9 h; *P*<0.001) and NaCl-lacking LB *(*barA/wild-type ratio of 0.25 at 9 h; *P*<0.001) (Fig 3B). Consistent with this observation, the ratio of phosphorylated RcsB (RcsB-P) to unphosphorylated RcsB was lower in the *barA* mutant than in wild-type *Salmonella* when bacteria were grown in either LB or NaCl-lacking LB to late exponential phase (OD_600_ of ~1.0) (Fig 3C). Control experiments showed no RcsB phosphorylation in a strain expressing the RcsB D56Q variant, and hyper RcsB phosphorylation in a strain expressing the constitutive *rcsC11* allele (Fig 3C).

Fluorescence was lower in the *rcsC barA* and *rcsD barA* double mutants than in the *rcsC* and *rcsD* single mutants, respectively, both in LB and NaCl-lacking LB media (Fig 3B), in agreement with the behavior displayed on solid media (Fig 2). Fluorescence was slightly lower in the *barA rcsC rcsD* triple mutant than in the *rcsC rcsD* double mutant both in LB and NaCl-lacking LB media (*P*<0.001 for NaCl-lacking LB at 9 h; *P*<0.04 for LB at 9 h) (Fig 3B), thus recapitulating the results obtained on solid media (Fig 2).

Cumulatively, the results presented in this section establish that BarA is required for full RcsB activation in LB broth and that this activation is time dependent. As discussed below, BarA uses multiple mechanisms to increase the amount of active RcsB protein but does not appear to target other regulators of the same protein family.

### BarA activates RcsB via the SirA-activated regulatory RNAs CsrB and CsrC

To date, BarA’s effect on gene expression has been largely ascribed to the regulatory RNAs CsrB and CsrC, which are encoded by genes directly activated by BarA’s cognate regulator SirA [23]. CsrB and CsrC act by sequestering CsrA, an RNA-binding protein that binds to the 5’ untranslated region (UTR) of hundreds of transcripts, typically decreasing their translation [29–32]. That BarA activation of RcsB may be mediated by CsrB and CsrC in *Salmonella* is also supported by the phenotype of a *csrB* mutant *Yersinia pseudotuberculosis* grown on solid media, which produced 8-fold less RcsB protein than the wild-type strain [33].

**Fig 4:**
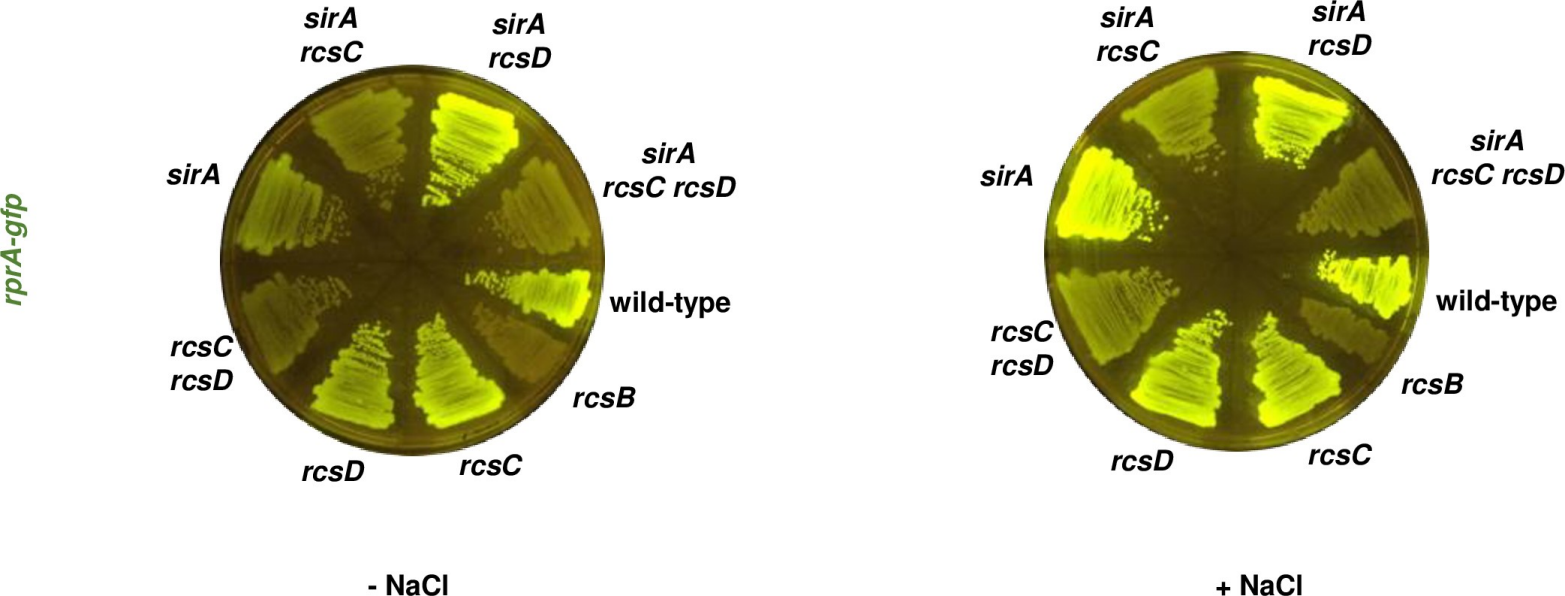
BarA activates RcsB via its cognate response regulator SirA. Fluorescence from wild-type (14028s), *rcsB* (EG12925), *rcsC* (HS1350), *rcsD* (HS1382), *rcsC rcsD* (HS1383), *sirA* (HS1565), *sirA rcsC* (HS1566), *sirA rcsD* (HS1567) and *sirA rcsC rcsD* (HS1568) *Salmonella* harboring plasmid pRprA-GFP (*rprA-gfp*) following 24 h of growth on LB solid medium without (-NaCl) or with (+NaCl) NaCl. Data are representative of two independent experiments, which gave similar results.

**Fig 5:**
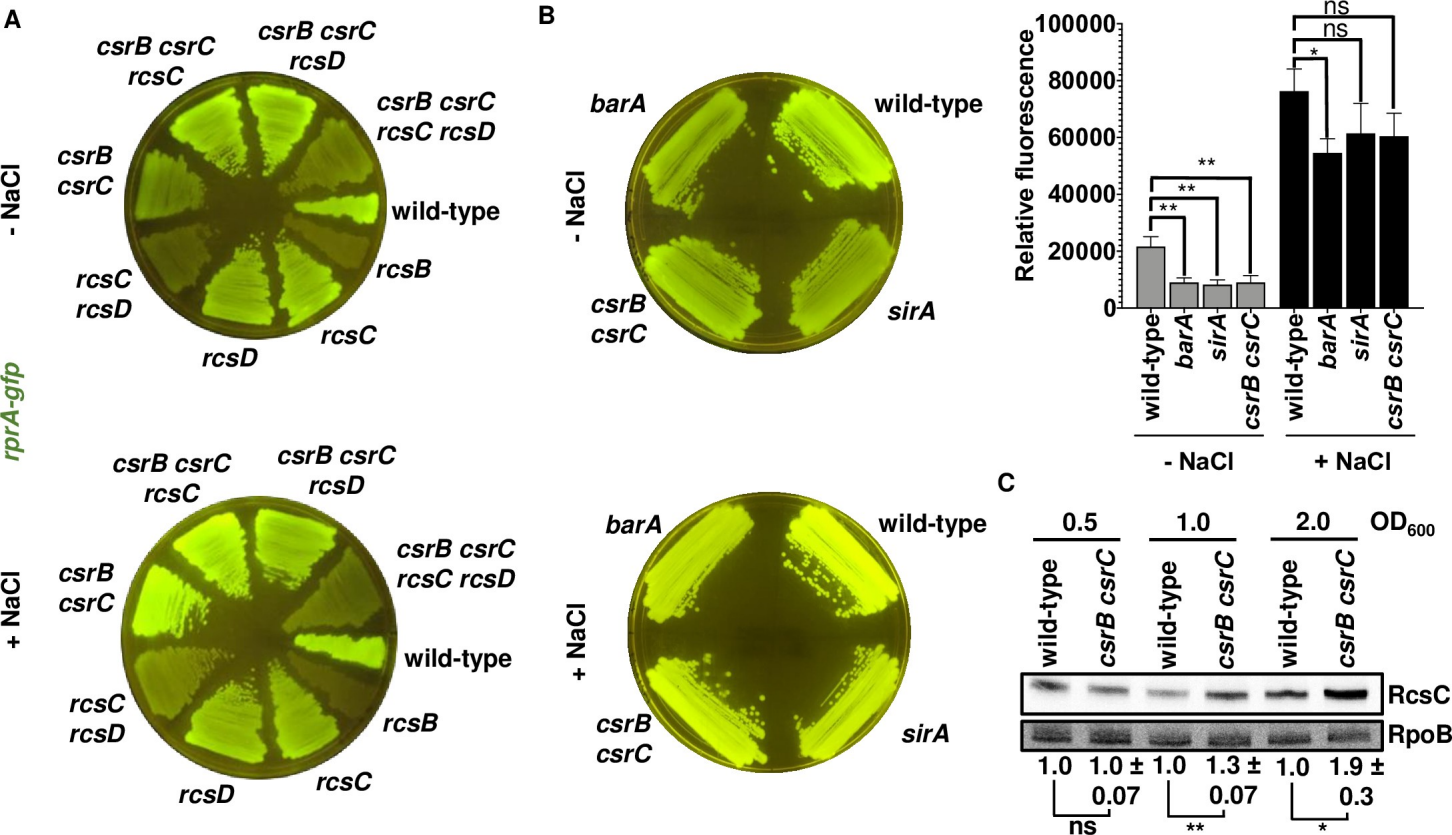
BarA activation of RcsB via the SirA-activated regulatory RNAs CsrB and CsrC. (A) The regulatory RNAs CsrB and CsrC are required for full RcsB activation on LB agar plates. Fluorescence from wild-type (14028s), *rcsB* (EG12925), *rcsC* (HS1350), *rcsD* (HS1382), *rcsC rcsD* (HS1383), *csrB csrC* (HS1651), *csrB csrC rcsC* (HS1654), *csrB csrC rcsD* (HS1655) and *csrB csrC rcsC rcsD* (HS1656) *Salmonella* harboring plasmid pRprA-GFP (*rprA-gfp*) following 24 h of growth on LB solid medium without (-NaCl) or with (+NaCl) NaCl. Data are representative of two independent experiments, which gave similar results. (B) Fluorescence from wild-type (14028s), *barA* (HS1520), *sirA* (HS1565) and *csrB csrC* (HS1651) *Salmonella* harboring plasmid pRprA-GFP (*rprA-gfp*) following 24 h of growth on LB solid medium without (-NaCl) or with (+NaCl) NaCl. Data are representative of three independent experiments, which gave similar results. Quantification of the fluorescence is provided next to the plate images. Values derived from three independent experiments (mean ± standard deviation) were statistically analyzed by Prism 8 using two-tailed unpaired *t* test. Statistical significance is indicated by * *P*<0.05, **P<0.01; ns, not significant. Error bars indicate standard deviation. (C) Western blot analysis of crude extracts from *rcsC-3XFLAG* (HS539) and *rcsC-3XFLAG csrB csrC* (HS2263) strains grown in LB NaCl-free broth. Samples were analyzed with antibodies directed to the FLAG epitope or the RpoB protein. Data are representative of three independent experiments, which gave similar results. RcsC levels for *csrB csrC* mutant strain relative to wild-type *Salmonella* are marked below (fold increase ± standard deviation). Values were statistically analyzed by Prism 8 using two-tailed unpaired *t* test. Statistical significance is indicated by * *P*<0.05, ** *P*<0.01; ns, not significant.

A *sirA* single mutant and a *csrB csrC* double mutant displayed half of the fluorescence of wild-type *Salmonella* when harboring the plasmid with the *rprA-gfp* fusion following growth on NaCl-lacking LB plates (Fig 4, Fig 5A and Fig 5B), behaving like the *barA* mutant (Fig 5B). By contrast, the three mutants behaved similarly to the wild-type strain in regular LB plates (Fig 3, Fig 4, Fig 5A and Fig 5B).

CsrB (and likely CsrC) is largely responsible for the BarA- and SirA-dependent activation of RcsB because a plasmid expressing CsrB from a heterologous promoter complemented not only the *csrB csrC* double mutant but also the *sirA* and *barA* single mutants (S4 Fig). This plasmid also increased the fluorescence of wild-type *Salmonella* (S4 Fig). By contrast, the vector control did not alter the fluorescence of any these strains. The CsrB-expressing plasmid did not increase the fluorescence of the RcsB D56Q variant (S5 Fig), indicating that CsrB’s action requires RcsB’s site of phosphorylation.

RcsC amounts were higher in the *csrB csrC* double mutant strain than in wild-type *Salmonella* at OD_600_ of 1.0 (a 1.3-fold difference) and 2.0 (a 1.9-fold difference) in LB broth without NaCl (Fig 5C). There were similar RcsC amounts at OD_600_ of 0.5, indicating that the negative regulation that CsrB and CsrC exert on RcsC is dependent on the growth phase. As a *rcsC* single mutant exhibited increased RcsB activity compared to wild-type *Salmonella* under the same experimental conditions (Fig 3B, left panel), one may expect CsrB and CsrC to activate RcsB under low osmolarity conditions by reducing RcsC expression. This is consistent with the observation that the *rcsC* single mutant did not exhibit reduced activity as compared to *rcsC csrB csrC* mutant on LB plates without NaCl (Fig 5A and Fig 5B). Moreover, a CsrB-expressing plasmid did not increase the fluorescence of a *rcsC csrB csrC* mutant (S6 Fig). This is in contrast to the higher fluorescence displayed by the *csrB csrC* double mutant when carrying pCsrB versus the pVector control (S6 Fig).

By contrast, RcsB amounts were the same for both wild-type *Salmonella* and the *csrB csrC* double mutant in LB broth without NaCl (S7A Fig), which is in contrast to previous findings reported with *Yersinia pseudotuberculosis* [33], and this was also the case for RcsD amounts (S7B Fig). Cumulatively, the results in this section strongly suggest that when wild-type *Salmonella* experiences low osmolarity, BarA activates RcsB-dependent transcription by reducing RcsC abundance through the action of the SirA-dependent CsrB and CsrC regulatory RNAs.

### SirA promotes *rcsDB* transcription from a *rcsD* promoter

When grown in LB without NaCl, the fluorescence of wild-type *Salmonella* harboring the plasmid with the *rprA-gfp* fusion was higher in cells harboring the plasmid expressing CsrB from a heterologous promoter than with the vector control (S4 Fig). This raised the possibility of the restoration of fluorescence conferred by the CsrB-expressing plasmid upon the *sirA* and *barA* mutants hiding other potential mechanisms by which the SirA and BarA proteins activate the RcsB protein. To explore this possibility, we examined fluorescence from the *rprA-gfp* fusion in isogenic strains with mutations in the *csrB* and *csrC* genes.

The *csrB csrC rcsC* and *csrB csrC rcsD* triple mutants had higher fluorescence than the *rcsC* and *rcsD* single mutants, respectively, on NaCl-lacking LB plates, which is in contrast to the lower fluorescence of the *csrB csrC* double mutant relative to wild-type *Salmonella* (Fig 5A, S8 Fig and S12 Fig). These results indicate that RcsB activation by CsrB and CsrC requires the RcsC and RcsD proteins. By contrast, the *sirA rcsC* mutant strain showed less fluorescence than the *csrB csrC rcsC* triple mutant (Fig 5A and S8 Fig) or the *rcsC* single mutant (Fig 5 and S8 Fig). Thus, SirA appears to activate RcsB independently of CsrB and CsrC.

**Fig 6:**
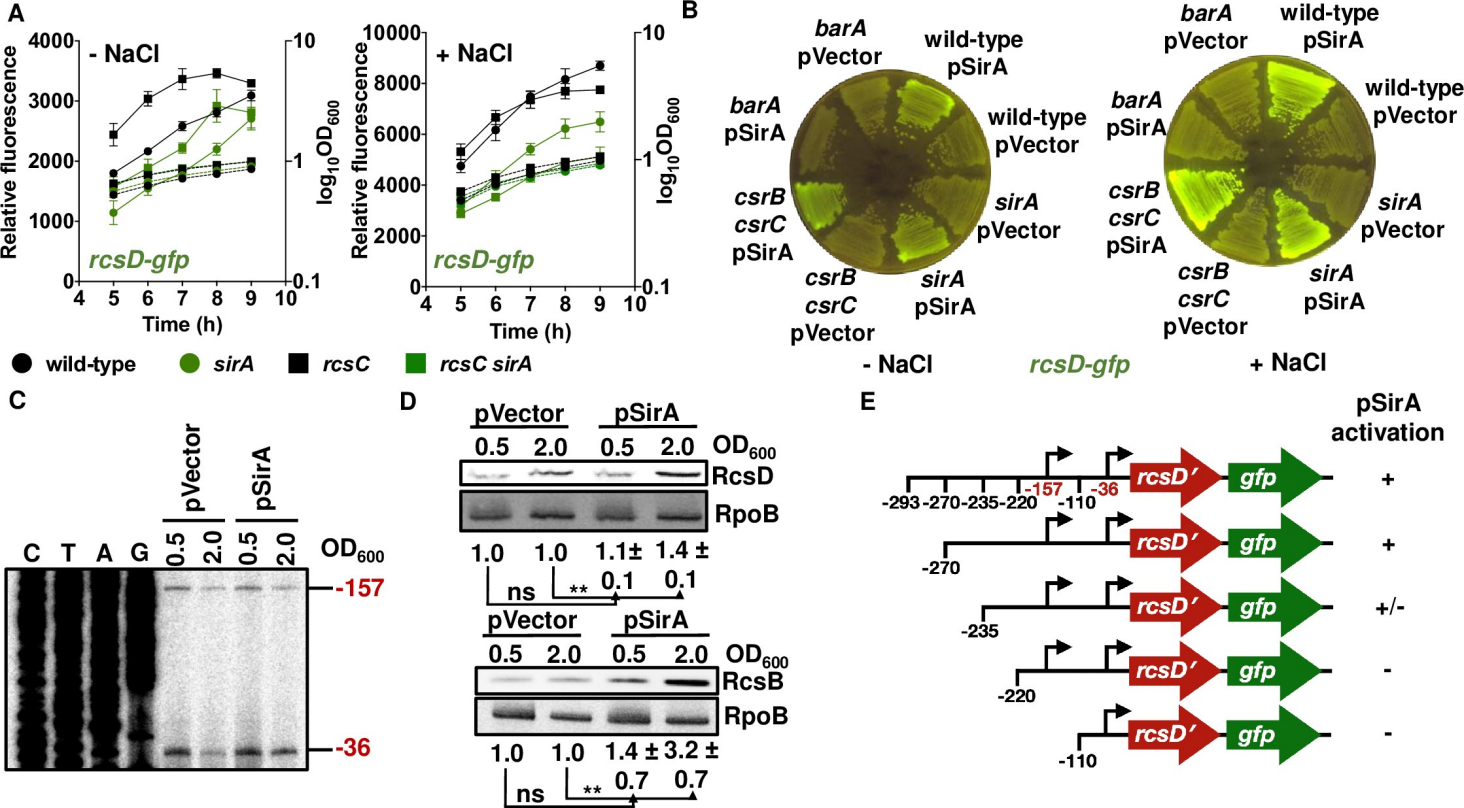
SirA promotes *rcsDB* transcription. (A) SirA is required for full *rcsDB* transcription. Fluorescence from wild-type (14028s), *sirA* (HS1565), *rcsC* (HS1350) and *rcsC sirA* (HS1566) *Salmonella* harboring pRcsD_-293_-GFP (*rcsD-gfp*) or pVector (empty pFPV25 vector) following 9 h of growth in LB liquid medium without (-NaCl) or with (+NaCl) NaCl. For each strain, relative fluorescence was obtained by subtracting the fluorescence of the strain harboring pVector from the fluorescence of the strain harboring pRcsD-GFP. The value obtained was then divided by OD_600_. Error bars represent standard deviation from three independent experiments. Values derived from three independent experiments (mean ± standard deviation) were statistically analyzed by Prism 8 using two-tailed unpaired *t* test. Significance values (*P*) are reported in the text. Relative fluorescence values (left axis) are represented by solid lines and OD_600_ values (right axis) by dotted lines. (B) SirA-mediated activation of *rcsDB* transcription requires BarA and occurs independently of CsrB and CsrC. Fluorescence from wild-type (14028s), *barA* (HS1520), *sirA* (HS1565) and *csrB csrC Salmonella* harboring pRcsD_-293_-GFP (*rcsD-gfp*) with pSirA or pVector (empty pACYC184 vector) following 24 h of growth on LB solid medium without (-NaCl) or with (+NaCl) NaCl. Data are representative of two independent experiments, which gave similar results. (C) Primer extension analysis of *rcsDB*_*-36*_ and *rcsDB*_*-157*_ levels in wild-type (14028s) *Salmonella* harboring pSirA or pVector (empty pACYC184 vector) grown in LB broth. Primer extension reaction was carried out on total RNA samples using primer W4171. (D) Western blot analysis of crude extracts from *rcsD-HA* (HS1309) and *rcsB-FLAG* (HS717) *Salmonella* harboring pSirA or pVector (empty pACYC184 vector) grown in LB broth. Samples were analyzed with antibodies directed to the FLAG or HA epitopes or the RpoB protein. Data are representative of three independent experiments, which gave similar results. RcsB and RcsD levels for pSirA strain relative pVector strain are marked below. Values were statistically analyzed by Prism 8 using two-tailed unpaired *t* test. Statistical significance is indicated by * *P*<0.05, ** *P*<0.01; ns, not significant. (E) Identification of the region upstream of the *rcsD* coding region required for SirA-mediated activation of *rcsDB*. Fluorescence from wild-type (14028s) *Salmonella* harboring pRcsD_-293_-GFP (*rcsD-gfp*), pRcsD_-270_-GFP, pRcsD_-235_-GFP, pRcsD_-220_-GFP or pRcsD_-110_-GFP with pSirA or pVector (empty pACYC184 vector) following 24 h of growth on LB solid medium without (-NaCl) or with (+NaCl) NaCl was monitored. SirA activation is defined by increased fluorescence in the pSirA strain as compared to the pVector strain (see S10 Fig for plates images). Plus sign (+) indicates fusion activation, plus/minus sign (+/-) indicates weak fusion activation, and minus sign (-) indicates no activation. The numbers refer to locations relative to the *rcsD* start codon. Data are representative of two independent experiments, which gave similar results.

Because the *rcsC sirA* and *rcsC rcsD* double mutants exhibited similar fluorescence from *rprA-gfp* (Fig 4), and because SirA is reported to act primarily as a transcriptional regulator [23, 25], we reasoned that SirA controls RcsD abundance. In support of this notion, the fluorescence of the *sirA* and *sirA rcsC* mutants strains was three-quarters to two-thirds that of wild-type *Salmonella* and of the *rcsC* mutant, respectively, (*P*<0.01 for *sirA* versus wild-type; *P*<0.001 for *sirA rcsC* versus *rcsC* at 7 h in NaCl-lacking LB; *P*<0.001 for *sirA* versus wild-type; *P*<0.001 for *sirA rcsC* versus *rcsC* at 7 h in LB) when carrying plasmid with a transcriptional fusion between the *rcsD* promoter and a promoterless *gfp* gene (Fig 6A).

When tested on LB plates, wild-type *Salmonella* and the *sirA* mutant, both carrying a plasmid expressing *sirA* from a heterologous promoter and the *rcsD-gfp* fusion plasmid, showed higher fluorescence than the corresponding strains harboring the vector control (Fig 6B). However, this was not the case for the *barA* mutant (Fig 6B), arguing that SirA activation is strictly dependent on BarA under the investigated conditions. By contrast, the *sirA*-expressing plasmid, but not the vector control, increased the fluorescence of the *csrB csrC* double mutant (Fig 6B). The SirA-mediated activation of the *rcsD* promoter occurs independently of RcsB, a negative regulator of the *rcsDB* operon [34], as pSirA increased the fluorescence from *rcsD-gfp* in the *rcsB* mutant (S9 Fig). Collectively, these results indicate that SirA activates the *rcsD* promoter independently of the CsrB and CsrC regulatory RNAs and of RcsB, and that this activation relies on SirA’s cognate phosphorelay sensor BarA.

To explore how SirA increases RcsD abundance, we mapped the *rcsD* transcription start site(s) in wild-type *Salmonella* harboring pSirA versus the pVector following growth in LB broth to an OD_600_ of 2.0. Two transcription start sites were identified 157 (*rcsDB*_-157_) and 36 (*rcsDB*_-36_) nucleotides upstream of the *rcsD* start codon (Fig 6C), in agreement with previous reports [34–36]. The abundance of the *rcsDB*_-36_ transcript was higher in the pSirA-containing strain than in the pVector-containing strain (Fig 6C). The SirA-dependent effect is growth phase specific because the isogenic strains behaved similarly when bacteria were harvested at an OD_600_ of 0.5 (Fig 6C). In contrast to the behavior of the *rcsDB*_-36_ transcript, the abundance of the *rcsD*_-157_ transcript was the same in the pSirA- and pVector-carrying strains at both optical densities (Fig 6C). The SirA-dependent increase in mRNA abundance from the *rcsDB*_-36_ transcript resulted in larger amounts of the RcsD (1.4-fold increase) and RcsB (3.2-fold increase) proteins (Fig 6D). Therefore, by promoting transcription from the *rcsDB*_-36_ start site, SirA increases the amounts of the *rcsDB* polycistronic transcript, resulting in higher amounts of the RcsD and RcsB proteins.

To identify *rcsDB* promoter element(s) required for SirA-mediated transcription, we examined the fluorescence of wild-type *Salmonella* harboring the *sirA-*expressing plasmid or the vector control, and a set of isogenic plasmids with an *rcsD-gfp* transcriptional fusion and various lengths of the putative *rcsD* promoter region (i.e. positions -270, -235, -220 and -110 relative to *rcsD* start codon) (Fig 6E). No SirA-dependent activation was observed in strains harboring the *rcsD*_-110_ and *rcsD*_-220_ fusions (Fig 6E and S10 Fig), similarly high activation with the *rcsD*_-270_ and *rcsD*_-293_ fusions (Fig 6E and S10 Fig), and intermediate activation with the *rcsD*_-235_ fusion (Fig 6E and S10 Fig). Thus, sequence elements located between positions -270 and -220 (i.e. 234 nts upstream the SirA-activated *rcsDB*_-36_ transcription start site) (S11 Fig) are required for SirA-mediated activation of *rcsDB*. These results are concordant with the presence of SirA binding sites in *csrB* and *csrC* promoters 215 and 181 nts upstream of the *csrB* and *csrC* transcription starts sites, respectively [23].

### BarA activates RcsB independently of the SirA, RcsC, and RcsD proteins, and of acetyl phosphate

**Fig 7:**
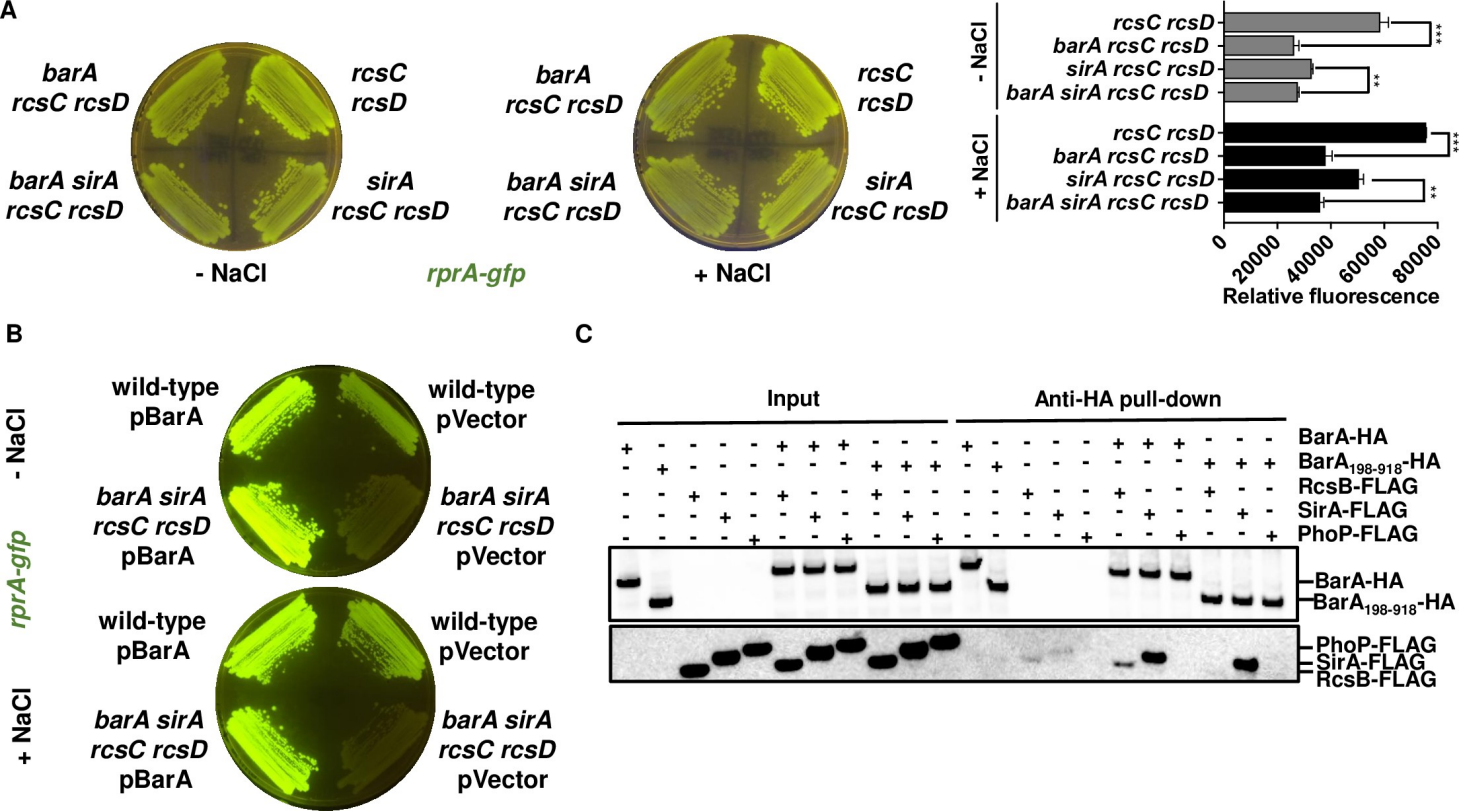
BarA activates RcsB independently of the SirA, RcsC, and RcsD proteins. (A) Effect of *barA* inactivation on RcsB activity in a *rcsC rcsD sirA* background. Fluorescence from *rcsC rcsD* (HS1383), *barA rcsC rcsD* (HS1523), *sirA rcsC rcsD* (HS1568), *barA sirA rcsC rcsD* (HS1796) *Salmonella* harboring plasmid pRprA-GFP (*rprA-gfp*) following 24 h of growth on LB solid medium without (-NaCl) or with (+NaCl) NaCl. Quantification of fluorescence is provided in the right panel of the figure. Values derived from three independent experiments (mean ± standard deviation) were statistically analyzed by Prism 8 using two-tailed unpaired *t* test. Statistical significance is indicated by **P<0.01, by ***P<0.001; ns, not significant. Error bars indicate standard deviation. (B) Heterologous expression of the *barA* gene recovers wild-type levels of fluorescence in a *barA sirA rcsC rcsD* strain. Fluorescence from wild-type (14028s) and HS1796 (*barA sirA rcsC rcsD*) *Salmonella* harboring plasmid pRprA-GFP (*rprA-gfp*) with pBarA or pVector (empty pACYC184 vector) following 24 h of growth on LB solid medium without (-NaCl) or with (+NaCl) NaCl. Data are representative of two independent experiments, which gave similar results. (C) The BarA and RcsB proteins interact. Anti-HA pull-down assay showing interactions between *in vitro*–synthesized BarA-HA, BarA_198-918_-HA, RcsB-FLAG, SirA-FLAG and PhoP-FLAG proteins. Samples were analyzed by Western blotting using antibodies recognizing the HA or FLAG epitopes.

Unexpectedly, the *barA rcsD* double mutant exhibited much lower fluorescence from *rprA-gfp* than the *sirA rcsD* double mutants in both LB and NaCl-lacking LB plates (S12 Fig). This result suggests that BarA can activate RcsB by a pathway not involving SirA. In agreement with this notion, a *barA sirA rcsC rcsD* quadruple mutant strain displayed slightly lower fluorescence from *rprA-gfp* than the *sirA rcsC rcsD* triple mutant in both LB and NaCl-lacking LB plates (Fig 7A). The BarA-expressing plasmid complemented the *barA sirA rcsC rcsD* quadruple mutant, whereas the vector control (pVector) did not (Fig 7B). Cumulatively, these results argue that BarA is capable of activating RcsB in a SirA-independent fashion.

BarA appears to activate RcsB directly because immunoprecipitation experiments using BarA-HA and *in vitro* synthesized proteins showed that RcsB-FLAG was pulled down by antibodies recognizing the HA epitope, indicating that BarA binds to RcsB (Fig 7C). The antibodies recognizing the HA epitope also pulled down SirA-FLAG, which was used as a positive control, but not the response regulator PhoP-FLAG, which was used as a negative control (Fig 7C). These *in vitro* results demonstrate that BarA binds to RcsB as well as to SirA.

Next, we examined the ability of BarA to autophosphorylate from adenosine triphosphate (ATP) and to serve as phosphodonor to RcsB *in vitro*. For these experiments, we used the BarA_198-918_ variant because the equivalent *E*. *coli* variant does autophosphorylate and serve as phosphodonor to its cognate regulator SirA, and also because, lacking amino acids 1–197 corresponding to the transmembrane segments, BarA_198-918_ is more suitable for purification than the full-length BarA protein [5]. The *Salmonella* BarA_198-918_ autophosphorylated and served as phosphodonor to the *Salmonella* SirA protein (S13 Fig), recapitulating the behavior reported for the *E*. *coli* sequelogs [5]. By contrast, RcsB was not phosphorylated from BarA_198-918_, behaving like the negative control PhoP (S13 Fig). Consistent with this result, a BarA_198-918_-expressing plasmid (pBarA_198-918_) showed a reduced ability to increase fluorescence from *rprA-gfp* in wild-type *Salmonella*, and to rescue the *rcsC rcsD sirA barA* quadruple mutant as compared to the strains expressing the full-length BarA (S15 Fig). The BarA_198-918_ variant does not retain the functionality of the full-length BarA because BarA_198-918_-HA bound SirA-FLAG but not RcsB-FLAG *in vitro* (Fig 7C). This result may account for the inability of BarA_198-918_ to serve as phosphodonor do RcsB *in vitro*. However, the pBarA_198-918_ plasmid promoted low level of RcsB activation in the quadruple *barA sirA rcsC rcsD* mutant strain (S15 Fig), leaving open the possibility of BarA promoting RcsB phosphorylation by a mechanism that does not require these two proteins to interact.

Given that RcsB can autophosphorylate from acetyl-phosphate (AcP), resulting in transcription of RcsB-activated genes [37, 38], and that BarA_198-918_ failed to promote RcsB phosphorylation *in vitro* (S13 Fig), we explored the possibility of BarA promoting RcsB phosphorylation from acetyl-phosphate. However, an *ackA-pta* mutant, which lacks the ability to synthesize acetyl-phosphate, exhibited the same fluorescence from *rprA-gfp* as wild-type *Salmonella* on LB plates with and without NaCl (S14 Fig). In addition, the *ackA pta barA* triple mutant was less fluorescent than the *ackA pta* double mutant on NaCl-lacking LB (S14 Fig), recapitulating the difference in fluorescence that exists between the *barA* mutant and wild-type *Salmonella* (S14 Fig). These data indicate that acetyl-phosphate does not participate in the BarA activation of RcsB under the investigated conditions.

### Agents that damage the outer membrane or cell wall activate RcsB in a BarA-independent manner

**Fig 8:**
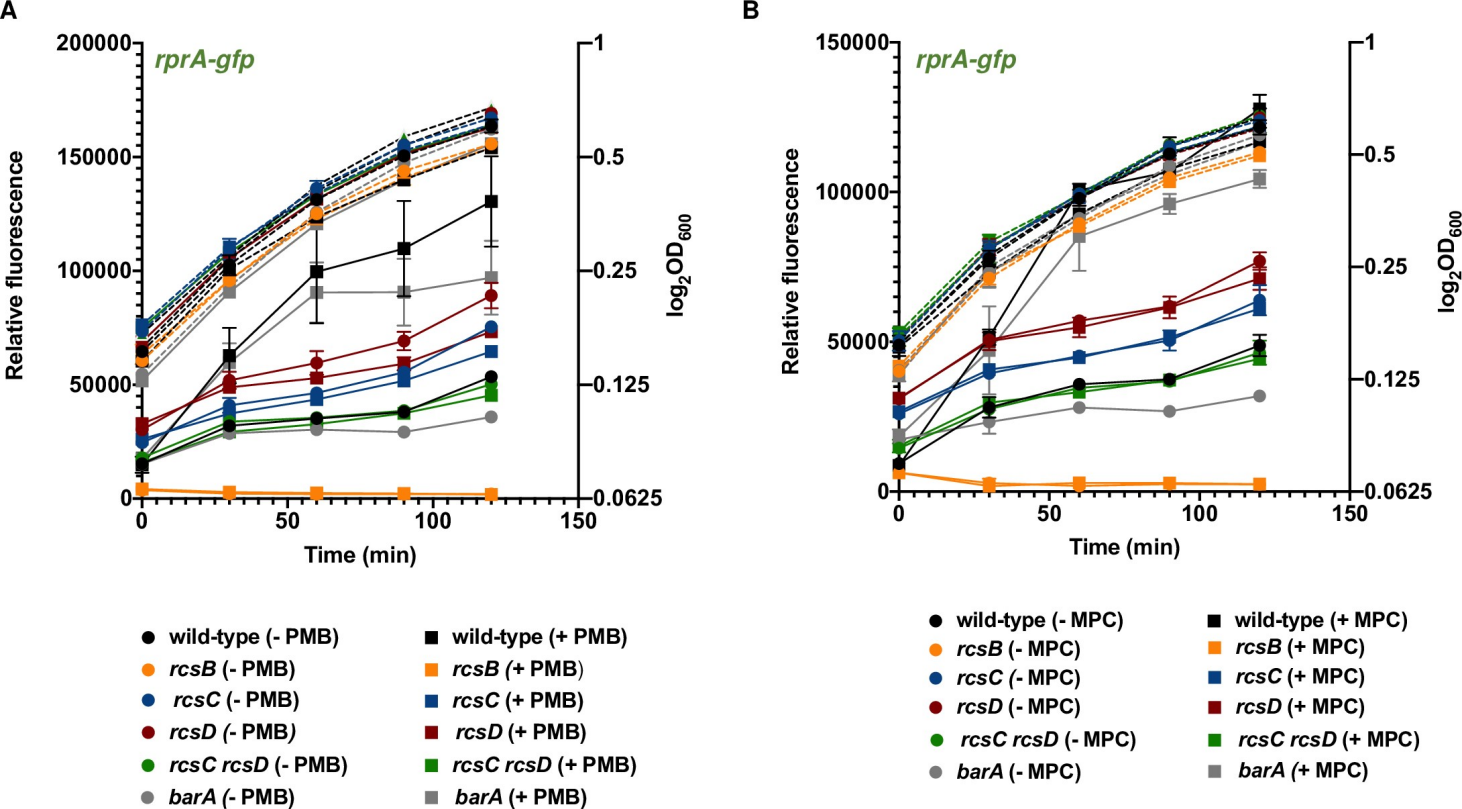
BarA is dispensable for RcsB activation upon damage of the outer membrane or cell wall. (A) Fluorescence from wild-type (14028s), *rcsB* (EG12925), *rcsC* (HS1350), *rcsD* (HS1382), *rcsC rcsD* (HS1383) and *barA* (HS1520) *Salmonella* harboring plasmid pRprA-GFP (*rprA-gfp*) or pVector (empty pFPV25 vector) upon 2 h treatment with a sublethal concentration (0.5 μg/ml) of polymyxin B (+ PMB) or with water (-PMB) in LB liquid medium. Polymyxin B was added in early exponential phase (OD_600_ of ~0.15). Relative fluorescence values (left axis) are represented by solid lines and OD_600_ values (right axis) by dotted lines. Values derived from three independent experiments (mean ± standard deviation) were statistically analyzed by Prism 8 using two-tailed unpaired *t* test. Significance values (*P*) are reported in the text. (B) Fluorescence from wild-type (14028s), *rcsB* (EG12925), *rcsC* (HS1350), *rcsD* (HS1382), *rcsC rcsD* (HS1383) and *barA* (HS1520) *Salmonella* harboring plasmid pRprA-GFP (*rprA-gfp*) or pVector (empty pFPV25 vector) upon 2 h treatment with a sublethal concentration (10 μg/ml) of mecillinam (+ MPC) or with water (-MPC) in LB liquid medium. Mecillinam was added in early exponential phase (OD_600_ of ~0.15). For each strain, relative fluorescence was obtained by subtracting the fluorescence of the strain harboring pVector from the fluorescence of the strain harboring plasmid pRprA-GFP. The obtained value was then divided by OD_600_. Error bars represent standard deviation from three independent experiments. Relative fluorescence values (left axis) are represented by solid lines and OD_600_ values (right axis) by dotted lines. Values derived from three independent experiments (mean ± standard deviation) were statistically analyzed by Prism 8 using two-tailed unpaired *t* test. Significance values (*P*) are reported in the text.

Polymyxin B activates RcsB by damaging the outer membrane, and mecillinam by damaging the cell wall [11, 22, 39]. These chemicals activate RcsB in a BarA-independent manner because a similar increase in fluorescence was observed in wild-type and *barA Salmonella* carrying the *rprA*-*gfp* fusion plasmid treated with polymyxin B (2.4-fold increase for wild-type; *p*<0.01 and 2.7-fold increase for *barA*; *p*<0.01 after 2 h of treatment) or mecillinam (2.6-fold increase for wild-type; *p*<0.0001 and 3.2-fold increase for *barA*; *p*<0.0001 after 2 h of treatment) during exponential growth in LB broth (Fig 8A and 8B). The *rcsB*, *rcsC*, and *rcsD* single mutants, and the *rcsC rcsD* double mutants showed no significant increase in fluorescence upon addition of either agent (Fig 8A and 8B), in agreement with the notion that RcsB activation by polymyxin B and mecillinam is dependent on both the RcsC and RcsD proteins [22, 39]

### BarA is dispensable for activation of the regulators ArcA, OmpR, and PhoP under conditions in which it activates RcsB

BarA appears to specifically control the activity of the regulators SirA and RcsB in LB agar with or without NaCl. This is because similar fluorescence was displayed by wild-type and *barA* mutant *Salmonella* harboring transcriptional fusions between a promoterless *gfp* gene and the promoter of the ArcA-repressed *lldP* gene (S16 Fig). This was also true for strains harboring *gfp* transcriptional fusions to the PhoP-activated *rstA* gene (S17 Fig) and the OmpR-activated *ompC* gene (S18 Fig). The ArcA, PhoP, and OmpR proteins are response regulators of the same protein family as RcsB and SirA.

## Discussion

We have now established that the response regulator RcsB is activated by the non-cognate phosphorelay protein BarA (Fig 1) during rapid growth of *Salmonella* in LB media, (Fig 2 and Fig 3). BarA activates RcsB in multiple ways: (i) via its cognate response regulator SirA (Fig 4), which promotes transcription of the *rcsDB* operon, thus increasing the abundance of the RcsD and RcsB proteins (Fig 6); (ii) via the SirA-activated regulatory RNAs CsrB and CsrC, which decrease the amounts of the phosphorelay protein RcsC (Fig 5); and (iii) by binding RcsB (Fig 7C), which activates RcsB independently of the RcsC, RcsD, and SirA proteins (Fig 7A and 7B). By contrast, BarA is not required for RcsB activation taking place in bacteria experiencing outer membrane stress or cell wall perturbations (Fig 8), which requires RcsB’s cognate phosphorelay proteins RcsC and RcsD (Fig 8) [10]. Our findings indicate that different phosphorelay proteins can act on a given regulator, depending on the environment experienced by a bacterium (Fig 1).

BarA activation of RcsB appears to be largely mediated by the SirA-activated CsrB and CsrC regulatory RNAs. This is because a *csrB csrC* mutant strain behaved the same way as *barA* and *sirA* single mutant strains regarding activation of the RcsB-dependent *rprA-gfp* fusion (Fig 5A and Fig 5B). There are two non-mutually exclusive possibilities about how these two regulatory RNAs may increase RcsB activity. On the one hand, the *csrB csrC* double mutant has more RcsC protein that wild-type *Salmonella* when bacteria are grown in LB broth without NaCl (Fig 5C), suggesting CsrB and CsrC activate RcsB by decreasing RcsC amounts. The higher RcsB activity exhibited by a *rcsC* mutant strain as compared to wild-type *Salmonella* in NaCl-lacking LB broth (Fig 3B, left panel) may reflect that RcsC can act as a phosphatase that removes a phosphate from RcsB in the absence of RcsC-activating signals [10, 15, 21, 40]. Therefore, by decreasing RcsC amounts, CsrB and CsrC would reduce RcsB dephosphorylation, resulting in RcsB-dependent gene transcription.

The regulatory RNAs CsrB and CsrC operate by binding to the RNA binding protein CsrA, thereby decreasing the CsrA amounts available to control expression of target RNAs [41, 42]. Analysis of the *rcsC* 5’ leader region revealed a putative CsrA binding site upstream of the *rcsC* start codon (S19 Fig) that partly matches the CsrA consensus recognition sequence [43]. While this analysis suggests that CsrA may directly regulate *rcsC* translation, the *rcsC* mRNA was not identified as a CsrA target in a recent genome-wide mapping of CsrA binding sites in *Salmonella* [29]. That this study was conducted under different growth conditions (i.e., stationary phase in regular LB) from those in which the *csrB* and *csrC* mutations increase RcsC amounts (i.e., exponential and stationary phases in LB without NaCl) (Fig 5C) may explain why *rcsC* was not recovered as a CsrA target. Of course, it is also possible that CsrA promotes RcsC expression via a yet to be identified target.

On the other hand, many mRNAs encoding proteins involved in cell envelope integrity have been identified as CsrA targets in both *Salmonella* and *E*. *coli* [29, 44, 45]. By titrating CsrA away from these mRNAs, the regulatory RNAs CsrB and CsrC could then affect their translation, potentially increasing RcsB activity.

The full-length BarA protein bound to RcsB as well as to SirA *in vitro* (Fig 7C) and activated RcsB in the absence of SirA *in vivo* (Fig 7A and 7B). That BarA activation of RcsB requires RcsB’s site of phosphorylation (S3 Fig) suggests that BarA promotes the phosphorylated state of RcsB. However, a truncated BarA protein failed to bind (Fig 7C), and hence, to serve as phosphodonor to RcsB although it did so to SirA (S13 Fig). This raises the possibility that the full-length BarA protein is capable of promoting *in vitro* RcsB phosphorylation, as suggested by the increased ability of the pBarA plasmid to activate RcsB as compared to the pBarA_198-918_ plasmid (S15 Fig). One may also consider the activation of RcsB by BarA independently of RcsC, RcsD, and SirA proteins (Fig 7A and 7B) to be indirect (Fig 1), requiring a yet to be identified factor essential for BarA to promote RcsB phosphorylation.

That BarA activates RcsB when cells are rapidly growing in LB media suggests that some genes from the RcsB regulon fulfill physiological functions when *Salmonella* is not experiencing the cell envelope perturbations that activate RcsB via the RcsC and RcsD proteins. In agreement with this notion, BarA impacts RcsB activity during logarithmic growth in LB medium (Fig 3A, 3B and 3C) but not in response to outer membrane stress and cell perturbations (Fig 8).

Activation of a regulator by non-cognate phosphorelay proteins is not exclusive to RcsB. This is because the regulator OmpR is activated both by its cognate sensor EnvZ upon high osmolarity [46] and by the non-cognate sensor ArcB when *E*. *coli* experiences anaerobic conditions [47]. ArcB forms an anaerobiosis-responsive phosphorelay with its cognate regulator ArcA [48–50]. Thus, OmpR-regulated genes respond to both osmolarity and oxygen availability. Yamamoto and colleagues have also reported several cases of *in vitro* phosphorylation of regulators by non-cognate sensor kinases [51]. However, the incubation times used for the transphosphorylation reactions in this study do not allow us to determine if the observed phosphotransfers between non-cognate proteins are physiologically relevant, as demonstrated by Laub and colleagues [52].

Two-component system proteins are generally encoded in the same operon [6, 53, 54], allowing their co-expression and making interactions with non-cognate partners less likely [6, 53]. Intriguingly, the *rcsB*, *rcsC*, and *rcsD* genes are transcribed in distinct fashions [3, 20]. That is, the *rcsC* gene is convergently transcribed towards the *rcsB* gene, which is located downstream of *rcsD*. Although *rcsD* and *rcsB* are part of the same transcription unit, a separate promoter located within the *rcsD* coding region drives *rcsB* transcription under conditions distinct from those operating on the *rcsD* promoter [36]. Likewise, the BarA and SirA proteins are encoded by separate genes in all species reported to carry both *barA* and *sirA* [5, 23]. Given that BarA activates both SirA and RcsB (Fig 1), and that proteins constituting phosphorelays are often encoded by independently transcribed genes, they may be more prone to establish physiological interactions with additional partners. In line with this hypothesis, *Acinetobacter baumannii* and *Allochromatium vinosum* encode BarA but neither SirA nor RcsB [23], suggesting that BarA interacts with a yet to be identified regulator.

## Materials and methods

### Bacterial strains, plasmids, primers and growth conditions

Bacterial strains and plasmids used in this study are listed in the table in S1 Table; oligonucleotide sequences are presented in the table in S2 Table. Single gene knockouts and deletions were carried out as described [55]. Mutations generated by this approach were subsequently moved into clean genetic backgrounds via phage P22-mediated transduction as described [56]. Details of strain constructions are presented in *SI Materials and Methods*. Unless specified, bacteria were grown in LB medium (10 g/L NaCl) at 37˚C. When required, media were supplemented with ampicillin (50 μg/ml), chloramphenicol (20 μg/ml), kanamycin (50 μg/ml), tetracycline (10 μg/ml).

### Strain construction

Mutant strains were constructed by using the one-step inactivation method [55] with pKD3 or pKD4 plasmid DNA as template and the following primer pairs: W3556-W3557 for *rcsC*::Cm^R^, W4311-W4312 for *rcsD*::Km^R^, W4470-W4471 for *sirA*::Cm^R^, W4541-W4542 for *csrB*::Cm^R^, and W4545-W4546 for *csrC*::Km^R^. Cassette insertion was confirmed by PCR with W3522-W3523 for Δ*rcsC*::Cm^R^, W3246-W3247 for Δ*rcsD*::Km^R^, W4472-W4473 for Δ*sirA*::Cm^R^, W4543-W4544 for Δ*csrB*::Cm^R^, and W4547-W4548 for Δ*csrC*::Km^R^. Mutations generated by this approach were subsequently moved into wild-type *S*. *enterica* strain 14028s via phage P22-mediated transduction as described [56].

The *rcsC-3XFLAG*::Cm^R^, *rcsB-FLAG*::Cm^R^ and *rcsD-HA*::Cm^R^ strains were constructed by using the one-step inactivation method [55] using pKD3 plasmid DNA as template and the following primer pairs: W2504-W2505 for *rcsC-3XFLAG*::Cm^R^, W2895-W2541 for *rcsB-FLAG*::Cm^R^ and W4246-W2585 for *rcsD-HA*::Cm^R^. Cassette insertion was confirmed by PCR with W2506-W2507 for *rcsC-3XFLAG*::Cm^R^, W2542-W2543 for *rcsB-FLAG*::Cm^R^ and W2542-W2543 for *rcsD-HA*::Cm^R^. The *rcsC-3XFLAG*::Cm^R^, *rcsB-FLAG*::Cm^R^ and *rcsD-HA*::Cm^R^ alleles were subsequently moved into wild-type strain 14028s via phage P22-mediated transduction as described [56].

To generate the *rcsBD56Q* strain, a first PCR product was generated with primers W4387 and W4388 using the plasmid pSLC-242 [57] as a template. The resulting PCR product was then integrated into the chromosome of wild-type *S*. *enterica* (14028s) via the one-step inactivation method [55] using plasmid pKD46. Recombinant cells containing the insertion were selected on LB supplemented with 20 μg/ml chloramphenicol at 30˚C. This insertion was subsequently replaced via a second pKD46-mediated recombination of pre-annealed W4391 and W4392 primers into the chromosome. Cells were recovered for 3 h as described [57] and selected on N-minimal medium agar plates [58] containing 50 μM glutamate, 50 μM histidine, 50 μM leucine, 100 μM methionine, 100 μM glutamine, 10 mM MgCl_2_ and 30 mM rhamnose as the sole carbon source. The allele replacement was confirmed by PCR with primers W4389-W4390 followed by DNA sequencing.

To generate the Δ*rcsF*::Cm^R^ strain (HS1326), Δ*rcsF*::Cm^R^ was moved from EG14499 strain (Δ*rcsF*::Cm^R^) into wild-type strain 14028s via P22-mediated transduction as described [56].

To generate the Δ*barA*::Cm^R^ strain (HS1520), Δ*barA*::Cm^R^ was moved from EG16441 (Δ*barA*::Cm^R^) strain into wild-type strain 14028s via P22-mediated transduction as described [56].

To generate the Δ*barA* Δ*ackA-pta*::Cm^R^ strain (HS1987), Δ*ackA-pta*::Cm^R^ was moved from MP1238 (Δ*ackA-pta*::Cm^R^) into strain HS1564 (Δ*barA*) via P22-mediated transduction as described [56].

To generate HS1521 (Δ*barA*::Cm^R^ Δ*rcsC*), HS1522 (Δ*barA*::Cm^R^ Δ*rcsD*) and HS1523 (Δ*barA*::Cm^R^ Δ*rcsC* Δ*rcsD*) strains, Δ*barA*::Cm^R^ was moved from EG16441 (Δ*barA*::Cm^R^) strain to HS1350 (Δ*rcsC*), HS1382 (Δ*rcsD*) and HS1383 (Δ*rcsC* Δ*rcsD*) strains, respectively, via P22-mediated transduction as described [56].

To generate HS1566 (Δ*sirA*::Cm^R^ Δ*rcsC*), HS1567 (Δ*sirA*::Cm^R^ Δ*rcsD*), HS1568 (Δ*sirA*::Cm^R^ Δ*rcsC* Δ*rcsD*) and HS1590 (Δ*barA* Δ*sirA*::Cm^R^) strains, Δ*sirA*::Cm^R^ was moved from HS1565 (Δ*sirA*::Cm^R^) strain to HS1350 (Δ*rcsC*), HS1382 (Δ*rcsD*), HS1383 (Δ*rcsC* Δ*rcsD*) and HS1564 (Δ*barA*) strains, respectively, via P22-mediated transduction as described [56].

To generate HS1566 (Δ*sirA*::Cm^R^ Δ*rcsC*), HS1567 (Δ*sirA*::Cm^R^ Δ*rcsD*), HS1568 (Δ*sirA*::Cm^R^ Δ*rcsC* Δ*rcsD*) and HS1590 (Δ*barA* Δ*sirA*::Cm^R^) strains, Δ*sirA*::Cm^R^ was moved from HS1565 (Δ*sirA*::Cm^R^) strain to HS1350 (Δ*rcsC*), HS1382 (Δ*rcsD*), HS1383 (Δ*rcsC* Δ*rcsD*) and HS1564 (Δ*barA*) strains, respectively, via P22-mediated transduction as described [56].

To generate HS1651 (Δ*csrB*::Cm^R^ Δ*csrC*::Km^R^), Δ*csrB*::Cm^R^ was moved from HS1608 (Δ*csrB*::Cm^R^) to HS1609 (Δ*csrC*::Km^R^) via P22-mediated transduction as described [56].

To generate HS1654 (Δ*csrB*::Cm^R^ Δ*csrC*::Km^R^ Δ*rcsC*), HS1655 (Δ*csrB*::Cm^R^ Δ*csrC*::Km^R^ Δ*rcsD*) and HS1656 (Δ*csrB*::Cm^R^ Δ*csrC*::Km^R^ Δ*rcsC* Δ*rcsD*), Δ*csrC*::Km^R^ was first moved from HS1609 (Δ*csrC*::Km^R^) strain to HS1350 (Δ*rcsC*), HS1382 (Δ*rcsD*) and HS1383 (Δ*rcsC* Δ*rcsD*) strains, respectively, via P22-mediated transduction as described [56]. The Δ*csrB*::Cm^R^ allele was then moved from HS1608 (Δ*csrB*::Cm^R^) to each of the resulting strains via P22-mediated transduction.

To generate HS1778 (*rcsB-FLAG*::Cm^R^ Δ*csrB* Δ*csrC*), *rcsB-FLAG*::Cm^R^ was moved from HS717 (*rcsB-FLAG*::Cm^R^) to HS1722 (Δ*csrB* Δ*csrC*) via P22-mediated transduction as described [56].

To generate HS2263 (*rcsC-3XFLAG*::Cm^R^ Δ*csrB* Δ*csrC*), *rcsC-3XFLAG*::Cm^R^ was moved from HS539 (*rcsC-3XFLAG*::Cm^R^) to HS1722 (Δ*csrB* Δ*csrC*) via P22-mediated transduction as described [56].

To generate HS2276 (*rcsD-HA*::Cm^R^ Δ*csrB* Δ*csrC*), *rcsD-HA*::Cm^R^ was moved from HS1309 (*rcsD-HA*::Cm^R^) to HS1722 (Δ*csrB* Δ*csrC*) via P22-mediated transduction as described [56].

To generate HS1796 (Δ*barA* Δ*rcsC* Δ*rcsD* Δ*sirA*::Cm^R^), Δ*sirA*::Cm^R^ was moved from HS1565 (Δ*sirA*::Cm^R^) strain to HS1774 (Δ*barA* Δ*rcsC* Δ*rcsD*).

When required, pCP20 helper plasmid [55] was used to remove antibiotic-resistance markers.

### Construction of plasmids

To construct pRprA-GFP, primers W4304-W4305 were used to amplify *rprA* -160 to +12 region (relative to *rprA* transcription start site) using *Salmonella* 14028s genomic DNA as template. The resulting PCR product was digested with EcoRI and BamHI and ligated into pFPV25 plasmid DNA [59] digested with the same restriction enzymes. The ligation reaction was transformed into DH5α cells by electroporation. The identity of *rprA* insert was verified by DNA sequencing using primer W804.

To construct pRcsD_-293_-GFP, pRcsD_-270_-GFP, pRcsD_-235_-GFP, pRcsD_-220_-GFP and pRcsD_-110_-GFP, primers W3102-W3103 (-293), W4807-W3103 (-270), W4808-W3103 (-235), W4725-W3103 (-220) and W4726-W3103 (-110) were used to amplify *rcsD* -293 to +24, -270 to +24, -235 to +24, -220 to +24 and -110 to +24 regions (positions relative to *rcsD* ATG start codon), respectively, using *Salmonella* 14028s genomic DNA as template. The resulting PCR products were digested with EcoRI and BamHI and ligated into pFPV25 plasmid DNA [59] digested with the same restriction enzymes. The ligation reactions were transformed into DH5α cells by electroporation. The identity of *rcsD* inserts was verified by DNA sequencing using primer W804.

To construct pLldP-GFP, primers W4587-W4588 were used to amplify *lldP* -611 to -103 region (relative to *lldP* ATG translation start site) using *Salmonella* 14028s genomic DNA as template. The resulting PCR product was digested with EcoRI and BamHI and ligated into pFPV25 plasmid DNA [59] digested with the same restriction enzymes. The ligation reaction was transformed into DH5α cells by electroporation. The identity of *lldP* insert was verified by DNA sequencing using primer W804.

To construct pOmpC-GFP, primers W4817-W4818 were used to amplify *ompC* -330 to -71 region (relative to *ompC* ATG translation start site) using *Salmonella* 14028s genomic DNA as template. The resulting PCR product was digested with EcoRI and BamHI and ligated into pFPV25 plasmid [59] digested with the same restriction enzymes. The ligation reaction was transformed into DH5α cells by electroporation. The identity of *ompC* insert was verified by DNA sequencing using primer W804.

To construct pBarA, primers W4640-W4592 were used to amplify *barA* -18 to + 2763 region (relative to *barA* ATG start codon) using *Salmonella* 14028s genomic DNA as template. The resulting PCR product was digested with EcoRI and ligated into pACYC184 plasmid [60] digested with the same restriction enzyme. The ligation reaction was transformed into DH5α cells by electroporation. The identity of *barA* insert was verified by DNA sequencing using primers W4596-W4597.

To construct pBarA_198-918_, primers W4954-W4592 were used to amplify *barA* +594 to + 2763 region (relative to *barA* ATG start codon) using *Salmonella* 14028s genomic DNA as template. The resulting PCR product was digested with EcoRI and ligated into pACYC184 plasmid [60] digested with the same restriction enzyme. The ligation reaction was transformed into DH5α cells by electroporation. The identity of *barA*_*198-918*_ insert was verified by DNA sequencing using primers W4596-W4597.

To construct pSirA, primers W4593-W4595 were used to amplify *sirA* -19 to + 669 region (relative to *sirA* TTG start codon) using *Salmonella* 14028s genomic DNA as template. The resulting PCR product was digested with EcoRI and ligated into pACYC184 plasmid [60] digested with the same restriction enzyme. The ligation reaction was transformed into DH5α cells by electroporation. The identity of *sirA* insert was verified by DNA sequencing using primers W4596-W4597.

To construct pCsrB, primers W4717-W4718 were used to amplify *csrB* -9 to +407 region (relative to *csrB* transcription start site) using *Salmonella* 14028s genomic DNA as template. The resulting PCR product was digested with EcoRI and ligated into pACYC184 plasmid [60] digested with the same restriction enzyme. The ligation reaction was transformed into DH5α cells by electroporation. The identity of *csrB* insert was verified by DNA sequencing using primers W4596-W4597.

To construct pQE30-BarA_198-918_, primers W4703-W4704 were used to amplify the *barA*_*198-918*_ (*barA* coding region; amino acids 198 to 918) using *Salmonella* 14028s genomic DNA as template. The resulting PCR product was digested with BamHI and ligated into pQE30 Xa (Qiagen) digested with the same restriction enzyme. The ligation reaction was transformed into *E*. *coli* M15 cells by electroporation. The identity of *barA*_*198-918*_ insert was verified by DNA sequencing using primer W4711.

To construct pQE30-RcsB, primers W4705-W4706 were used to amplify the *rcsB* coding region using *Salmonella* 14028s genomic DNA as template. The resulting PCR product was digested with BamHI and ligated into pQE30 Xa (Qiagen) digested with the same restriction enzyme. The ligation reaction was transformed into *E*. *coli* M15 cells by electroporation. The identity of *rcsB* insert was verified by DNA sequencing using primer W4711.

To construct pQE30-SirA, primers W4707-W4708 were used to amplify the *sirA* coding region using *Salmonella* 14028s genomic DNA as template. The resulting PCR product was digested with BamHI and ligated into pQE30 Xa (Qiagen) digested with the same restriction enzyme. The ligation reaction was transformed into *E*. *coli* M15 cells by electroporation. The identity of *sirA* insert was verified by DNA sequencing using primer W4711.

To construct pQE30-PhoP, primers W4709-W4710 were used to amplify the *phoP* coding region using *Salmonella* 14028s genomic DNA as template. The resulting PCR product was digested with BamHI and ligated into pQE30 Xa (Qiagen) digested with the same restriction enzyme. The ligation reaction was transformed into *E*. *coli* M15 cells by electroporation. The identity of *phoP* insert was verified by DNA sequencing using primer W4711.

### Mutagenesis with transposon Tn10*d*Tc

Wild-type *Salmonella* 14028s was transformed with the Tn*10* transposase-expressing plasmid pNK972 [61]. A P22 lysate generated in strain TH338 (F::Tn*10d*Tc) was used to transduce the pNK972-carrying strain to randomly mutagenize the wild-type strain 14028s selecting for resistance to tetracycline. About 10,000 transductants were pooled together to generate a Tn*10d*Tc‐generated mutant library. The HS1383 strain (Δ*rcsC* Δ*rcsD*) carrying the pRprA-GFP plasmid was then transduced with the mutant library and about 9,000 transductants were screened for decreased fluorescence on LB agar plate. Two transductants displayed decreased fluorescence. The two transductants were infected with phage P22 and the the generated lysates were used to transduce HS1383 strain carrying plasmid pRprA-GFP. In both cases, the transductants displayed the phenotype of the original mutants. The identity of the genes responsible for the change in *rprA* GFP fusion activity, we determined the nucleotide sequence of the Tn*10d*Tc-chromosome joint as described [62] by performing a first PCR reaction with W4448-W4449 primers from genomic DNA extracted from the mutants with DNeasy Blood & Tissue Kits (Qiagen), and then a second PCR reaction using the product of the first reaction with the W4451-W4452 primers. The final PCR product was then sent for sequencing with W4451-W4452 primers.

### Colony plate fluorescence imaging and quantification

*Salmonella* cells expressing plasmid-borne *gfp* fusions were streaked onto either LB or on LB without NaCl agar plates supplemented with the appropriate antibiotics. Following 24 h of growth, fluorescence was visualized with a dark blue light transilluminator and an amber screen. For fluorescence quantification, isolated colonies were resuspended in 1 ml of phosphate-buffered saline (PBS) and 150 μl were aliquoted in a clear-bottomed 96-well black plates (Corning). Green fluorescence was measured using an Infinite M1000 plate reader (Tecan) with 485-nm excitation and 535-nm emission, and the absorbance was measured at 600 nm.

### Fluorescence measurement in liquid cultures

Overnight cultures of *Salmonella* in LB were diluted 1/1000 in LB or LB without NaCl supplemented with the appropriate antibiotics. Cells were grown in clear-bottomed 96-well black plates (Corning) using a SpectraMax Plus Microplate Reader (Molecular Devices) for agitation. Green fluorescence was measured using a Synergy H1 plate reader (BioTek) with 485-nm excitation and 535-nm emission, and the absorbance in each well was measured at 600 nm.

### Pull-down assays with proteins synthesized using an *in vitro* transcription-translation system

Pull-down assays were performed as previously described [63] with some modifications. Proteins were produced from DNA templates by *in vitro* synthesis using the PURExpress system (New England Biolabs). To synthesize the DNA templates, primers W4598-W4599 (*barA-HA*), W4798-W4599 (*barA*_*198-918*_*-HA*), W4608-W4609 (*rcsB-FLAG*), W4623-W4624 (*sirA-FLAG*), and W4621-W4622 (*phoP-FLAG*) were used. Synthesized proteins were mixed in 500 μl of tris-buffered saline (TBS) containing proteoliposomes (0.12 mg/ml) and incubated at room temperature for 2 h. Samples were then pulled-down with anti-HA magnetic beads (Thermo Scientific) at room temperature for 2 h. Samples were then analyzed by Western blot with antibodies directed to the FLAG (Abcam) or HA (Sigma) epitopes.

### Polymyxin B and mecillinam assays in liquid cultures

Overnight cultures of *Salmonella* strains in LB were diluted 1/1000 in LB supplemented with the appropriate antibiotics. Cells were grown into clear-bottomed 96-well black plates until early exponential phase (OD_600_ of ~0.15) and sublethal concentrations of polymyxin B (0.5 μg/ml) or mecillinam (10 μg/ml) were added. Water was added to untreated samples. Fluorescence was then monitored for a period of 2 h following the addition of the antimicrobial agents.

### Protein expression and purification

*E*. *coli* M15 cells co-transformed with pREP4 and the appropriate pQE30 derivatives expressing N-terminally His6-tagged proteins were grown in 250 ml of LB broth supplemented with 100 μg/ml ampicillin and 25 μg/ml kanamycin. Expression of the His6-tagged proteins was induced at mid-exponential phase (A_600nm_ of 0.5) by the addition of 2 mM isopropyl-β-D-thiogalactopyranoside (IPTG). Cultures were then grown at 30˚C for 4 h and cells were collected by centrifugation (5000 x *g* at 4˚C for 20 min). Pellets were then washed with 10 ml of cold PBS (5000 x *g* centrifugation at 4˚C for 20 min) and were stored at -20˚C. His6-tagged proteins were purified from cell pellets using Ni-NTA Fast Start Kit (Qiagen) according to the manufacturer’s instructions. The purified proteins were diluted 1/30 in 14 ml of kinase buffer (33 mM HEPES pH 7.5, 50 mM KCl, 5 mM MgCl_2_, 1 mM DTT, 0.1 mM EDTA, 10% glycerol) and were concentrated in Ultra-15 Centrifugal Filter Unit (Amicon). Protein concentrations were determined by direct A280 measurement of the concentrated samples.

### Phosphotransfer profiling

Phosphorylation assays were performed as previously described [5] with some modifications. Purified BarA protein (0.96 μM final) was first incubated in kinase buffer (33 mM HEPES pH 7.5, 50 mM KCl, 5 mM MgCl_2_, 1 mM DTT, 0.1 mM EDTA, 10% glycerol) in the presence of 15 Ci/mmol of [γ-^32^P]ATP (PerkinElmer) for 30 minutes at room temperature. Purified RcsB, SirA or PhoP proteins were then added to the mixture at final concentrations of 4 and 7 μM (final reaction volume of 10 μl) and the incubation was pursued for 15 min. Reactions were stopped by the addition of 10 μl of 2X LDS sample buffer and samples were run, without heating, on NuPAGE^TM^ 4–12% bis-tris protein gels (ThermoFisher Scientific). The gels were dried at 80˚C for 30 min and exposed to a phosphor screen overnight.

### Western blot assay

Cells were grown in LB broth media with or without NaCl. To extract total proteins, cells were precipitated with trichloroacetic acid (5% total volume) and washed with 80% acetone. Samples were resuspended in NuPAGE^TM^ LDS sample buffer (ThermoFisher Scientific) and normalized according to the OD_600_. Protein samples were run on NuPAGE^TM^ 4–12% bis-tris protein gels (ThermoFisher Scientific) and transferred to nitrocellulose membrane using iBlot Gel Transfer Device (ThermoFisher Scientific). Membranes were blocked with 5% milk solution in TBST for 1 h. Membranes were probed with 1:5000 dilution of mouse anti-FLAG (Sigma), rabbit anti-HA (Sigma) or mouse anti-RpoB (BioLegend). Secondary horseradish peroxidase-conjugated anti-rabbit (GE healthcare) or anti-mouse (Promega) was used at 1:5000 dilution. The blots were developed with the Amersham ECL Western blotting detection reagents (GE Healthcare) or SuperSignal West Femto chemiluminescent system (Pierce). Images were acquired with LAS-4000 imager (GE Healthcare). Images were quantified using ImageLabTM software (Biorad).

### *In vivo* detection of phosphorylated RcsB

Cells were grown in LB broth media with or without NaCl. Whole-cell extracts were prepared as previously described [64]. Samples were run on 12.5% polyacrylamide gels containing acrylamide–Phos-tag ligand (Wako Laboratory Chemicals) in standard running buffer [0.4% (w/v) SDS, 25 mM tris, 192 mM glycine] at 150 V at 4˚C for 4 h, transferred to nitrocellulose membranes, and analyzed by immunoblotting using polyclonal rabbit antibodies recognizing RcsB (a kind gift from Anna Vianney, Centre de Recherche en Infectiologie, INSERM, Lyon, France) (1:1000) and polyclonal mouse antibodies recognizing AtpB (Abcam) (1:5000). The blots were developed with the SuperSignal West Femto chemiluminescent reagents (Pierce). Images were acquired with LAS-4000 imager (GE Healthcare).

### Primer extension analysis

Cells were grown in LB broth and total RNA was extracted using the hot phenol procedure as previously described [65]. Primer extension reactions were then performed as previously described [66] using 20 μg of total RNA and the primer W4171 annealing with *rcsD* coding region. Primer extension reactions were run together with a template-specific sequencing ladder generated with primer W4171 and a DNA template corresponding to *rcsD* -228 to +38 region (relative to *rcsD* ATG start codon) amplified with primers W3228-W3229 using *Salmonella* 14028s genomic DNA as template.

## Supporting information

S1 Fig Transposon Tn*10d*Tc-generated mutagenesis identifies *barA* as an activator of RcsB. Fluorescence from *rcsC rcsD* (HS1383) *Salmonella* harboring plasmid pRprA-GFP (*rprA-gfp*) and of isogenic mutants with Tn*10d*Tc insertion in the *barA* and *rcsB* genes. The genomic location in the *Salmonella enterica* serovar Typhimurium 14028S genome of each Tn*10d*Tc insertion is indicated below each strain. A derivative of the *rcsC rcsD* parental strain with a Tn*10d*Tc is also shown.
(TIF)

S2 Fig *barA* expression from a heterologous promoter restores wild-type levels of fluorescence to a *barA* strain. Fluorescence from wild-type (14028s) and HS1520 (*barA*) *Salmonella* harboring plasmid pRprA-GFP (*rprA-gfp*) and pBarA or pVector (empty pACYC184 vector) following 24 h of growth on LB solid medium without (-NaCl) or with (+NaCl) NaCl. Data are representative of two independent experiments, which gave similar results.
(TIF)

S3 Fig BarA-mediated activation of *rprA-gfp* fusion requires RcsB’s phosphorylation site. Fluorescence from wild-type (14028s) and *rcsBD56Q* (HS1483) *Salmonella* harboring plasmid pRprA-GFP (*rprA-gfp*) with pBarA or pVector (empty pACYC184 vector) following 24 h of growth on LB solid medium without (-NaCl) or with (+NaCl) NaCl. Data are representative of two independent experiments, which gave similar results.
(TIF)

S4 Fig Heterologous expression of *csrB* recovers wild-type levels of fluorescence in *barA*, *sirA* and *csrB csrC* mutant strains. Fluorescence from wild-type (14028s), *barA* (HS1520), *sirA* (HS1565) and *csrB csrC* (HS1651) *Salmonella* harboring plasmid pRprA-GFP (*rprA-gfp*) with pCsrB or pVector (empty pACYC184 vector) following 24 h of growth on LB solid medium without (-NaCl) or with (+NaCl) NaCl. Data are representative of two independent experiments, which gave similar results.
(TIF)

S5 Fig CsrB-mediated activation of *rprA-gfp* fusion requires RcsB’s phosphorylation site. Fluorescence from wild-type (14028s) and *rcsBD56Q* (HS1483) *Salmonella* harboring plasmid pRprA-GFP (*rprA-gfp*) with pCsrB or pVector (empty pACYC184 vector) following 24 h of growth on LB solid medium without (-NaCl) or with (+NaCl) NaCl. Data are representative of two independent experiments, which gave similar results.
(TIF)

S6 Fig Heterologous expression of the *csrB* gene does not increase the fluorescence of a *rcsC csrB csrC* triple mutant. Fluorescence from *csrB csrC* (HS1651) and *csrB csrC rcsC* (HS1654), *Salmonella* harboring plasmid pRprA-GFP (*rprA-gfp*) with pCsrB or pVector (empty pACYC184 vector) following 24 h of growth on LB solid medium without (-NaCl) or with (+NaCl) NaCl. Data are representative of two independent experiments, which gave similar results.
(TIF)

S7 Fig The regulatory RNAs CsrB and CsrC do not affect RcsB and RcsD protein amounts. (A) Western blot analysis of crude extracts prepared from *rcsB-FLAG* (HS717) and *rcsB-FLAG csrB csrC* (HS1778) *Salmonella* grown in LB NaCl-free broth. Samples were analyzed with antibodies directed to the FLAG epitope or the RpoB protein. (B) Western blot analysis of crude extracts prepared from *rcsD-HA* (HS1309) and *rcsD-HA csrB csrC* (HS2276) *Salmonella* grown in LB NaCl-free broth. Samples were analyzed with antibodies directed to the HA epitope or the RpoB protein. Data are representative of two independent experiments, which gave similar results.
(TIF)

S8 Fig SirA activates RcsB independently of the regulatory RNAs CsrB and CsrC. Fluorescence from *rcsC* (HS1350), *barA rcsC* (HS1521), *sirA rcsC* (HS1566) and *csrB csrC rcsC* (HS1654) *Salmonella* harboring plasmid pRprA-GFP (*rprA-gfp*) following 24 h of growth on LB solid medium without (-NaCl) or with (+NaCl) NaCl. Data are representative of three independent experiments, which gave similar results. Quantification of the fluorescence is provided on the right panel of the figure. Values derived from three independent experiments (mean ± standard deviation) were statistically analyzed by Prism 8 using two-tailed unpaired *t* test. Statistical significance is indicated by *P<0.05, ** *P*<0.01, *** *P*<0.001, **** *P*<0.0001; ns, not significant. Error bars indicate standard deviation.
(TIF)

S9 Fig SirA activates *rcsD* independently of RcsB. Fluorescence from wild-type (14028s) and *rcsB* (EG12925) *Salmonella* harboring plasmid pRprA-GFP with pSirA or pVector (empty pACYC184 vector) following 24 h of growth on LB solid medium without (-NaCl) or with (+NaCl) NaCl. Data are representative of two independent experiments, which gave similar results.
(TIF)

S10 Fig Identification of the region upstream of the *rcsD* coding region required for SirA-mediated activation of *rcsDB*. Fluorescence from wild-type (14028s) harboring pRcsD_-293_-GFP (*rcsD-gfp*), pRcsD_-270_-GFP, pRcsD_-235_-GFP, pRcsD_-220_-GFP or pRcsD_-110_-GFP with pSirA or pVector (empty pACYC184 vector) following 24 h of growth on LB solid medium without (-NaCl) or with (+NaCl) NaCl was monitored. The numbers -293, -270, -235, -220 and -110 refer to locations relative to the *rcsD* start codon. Data are representative of two independent experiments, which gave similar results.
(TIF)

S11 Fig Identification of a putative SirA binding site in *rcsDB* promoter. Underlined nucleotides represent the putative SirA binding site based on the results of Fig 6 and S10 Fig. The numbers -293, -270, -235, -220 and -110 refer to locations relative to the *rcsD* start codon (indicated in bold green letters).
(TIF)

S12 Fig BarA activates RcsB independently of SirA. Fluorescence from *rcsD* (HS1382), *barA rcsD* (HS1522), *sirA rcsD* (HS1567) and *csrB csrC rcsD* (HS1655) *Salmonella* harboring pRprA-GFP (*rprA-gfp*) following 24 h of growth on LB solid medium without (-NaCl) or with (+NaCl) NaCl. Data are representative of three independent experiments, which gave similar results. Quantification of the fluorescence is provided on the right panel of the figure. Values derived from three independent experiments (mean ± standard deviation) were statistically analyzed by Prism 8 using two-tailed unpaired *t* test. Statistical significance is indicated by *P<0.05, ** *P*<0.01, *** *P*<0.001; **** *P*<0.0001; ns, not significant. Error bars indicate standard deviation.
(TIF)

S13 Fig Phosphotransfer between BarA and the SirA, RcsB or PhoP proteins. Purified BarA_198-918_ (0.96 μM final) was first incubated with [γ-^32^P]ATP for 30 minutes at room temperature. SirA, RcsB or PhoP were then added at the indicated concentrations and the incubation was pursued for 15 min at room temperature before being stopped by the addition of 2X LDS-sample buffer. Data are representative of two independent experiments, which gave similar results.
(TIF)

S14 Fig BarA activates RcsB independently of acetyl-phosphate. (A) Fluorescence from wild-type (14028s), *barA* (HS1564), *ackA-pta* (MP1238) and *barA ackA-pta* (HS1987) *Salmonella* harbouring plasmid pRprA-GFP following 24 h of growth on LB solid medium without (-NaCl) or with (+NaCl) NaCl. Data are representative of two independent experiments, which gave similar results.
(TIF)

S15 Fig The BarA_198-918_ variant has reduced ability in promoting RcsB activation. Fluorescence from wild-type (14028) and *rcsC rcsD sirA barA* (HS1796) *Salmonella* harboring pRprA-GFP (*rprA-gfp*) with pSirA or pVector (empty pACYC184 vector) following 24 h of growth on LB solid medium without (-NaCl) or with (+NaCl) NaCl. Data are representative of two independent experiments, which gave similar results.
(TIF)

S16 Fig BarA does not affect the expression of the ArcA-repressed *lldP*-*gfp* fusion. Fluorescence from wild-type (14028s), *rcsB* (EG12925), *barA* (HS1520), *arcA* (MK71) and *arcB* (EG16900) *Salmonella* harboring pLldP-GFP (*lldP*-*gfp*) following 24 h of growth on LB solid medium without (-NaCl) or with (+NaCl) NaCl. Data are representative of two independent experiments, which gave similar results.
(TIF)

S17 Fig BarA does not affect the expression of the PhoP-activated *rstA-gfp* fusion. Fluorescence from wild-type (14028s), *rcsB* (EG12925), *barA* (HS1520), *phoP* (MS7953s) *Salmonella* harboring pRstA-GFP (*rstA*-*gfp*) or pVector (empty pMS201) following 24 h of growth on LB solid medium without (-NaCl) or with (+NaCl) NaCl. Data are representative of two independent experiments, which gave similar results.
(TIF)

S18 Fig BarA does not affect the expression of the OmpR-activated *ompC-gfp* fusion. Fluorescence from wild-type (14028s), *rcsB* (EG12925), *barA* (HS1520), *ompR* (EG14379) *Salmonella* harboring pOmpC-GFP (*ompC*-*gfp*) following 24 h of growth on LB solid medium without (-NaCl) or with (+NaCl) NaCl. Data are representative of two independent experiments, which gave similar results.
(TIF)

S19 Fig Predicted CsrA binding site in the 5’-untranslated (UTR) region of *rcsC* mRNA. The CsrA binding site consensus sequence is shown above the predicted CsrA binding site in *rcsC* mRNA. Vertical lines mark the residues in the predicted site that match those in the consensus.
(TIF)

S1 Data Prism spreadsheet of the numerical values underlying the data presented in Fig 2. Statistical analysis details are also included.
(PZFX)

S2 Data Prism spreadsheet of the numerical values underlying the data presented in Fig 3B. Statistical analysis details are also included.
(PZFX)

S3 Data Prism spreadsheet of the numerical values underlying the data presented in Fig 5B. Statistical analysis details are also included.
(PZFX)

S4 Data Prism spreadsheet of the numerical values underlying the data presented in Fig 5C. Statistical analysis details are also included.
(PZFX)

S5 Data Prism spreadsheet of the numerical values underlying the data presented in Fig 6A. Statistical analysis details are also included.
(PZFX)

S6 Data Prism spreadsheet of the numerical values underlying the data presented in Fig 6D for the Western blot analysis of RcsB levels. Statistical analysis details are also included.
(PZFX)

S7 Data Prism spreadsheet of the numerical values underlying the data presented in Fig 6D for the Western blot analysis of RcsD levels. Statistical analysis details are also included.
(PZFX)

S8 Data Prism spreadsheet of the numerical values underlying the data presented in Fig 7A. Statistical analysis details are also included.
(PZFX)

S9 Data Prism spreadsheet of the numerical values underlying the data presented in Fig 8. Statistical analysis details are also included.
(PZFX)

S10 Data Prism spreadsheet of the numerical values underlying the data presented in S8 Fig. Statistical analysis details are also included.
(PZFX)

S11 Data Prism spreadsheet of the numerical values underlying the data presented in S12 Fig. Statistical analysis details are also included.
(PZFX)

S1 Table Bacterial strains and plasmids used in this study. (DOCX)

S2 Table Oligonucleotides sequences used in this study. (DOCX)
