# Supplementary figures and images for: The phosphorelay BarA/SirA activates the non-cognate regulator RcsB in Salmonella enterica
Source: PLoS Genet. 2020 May 11;16(5):e1008722. doi: 10.1371/journal.pgen.1008722 (PMC7241856; doi:10.1371/journal.pgen.1008722)

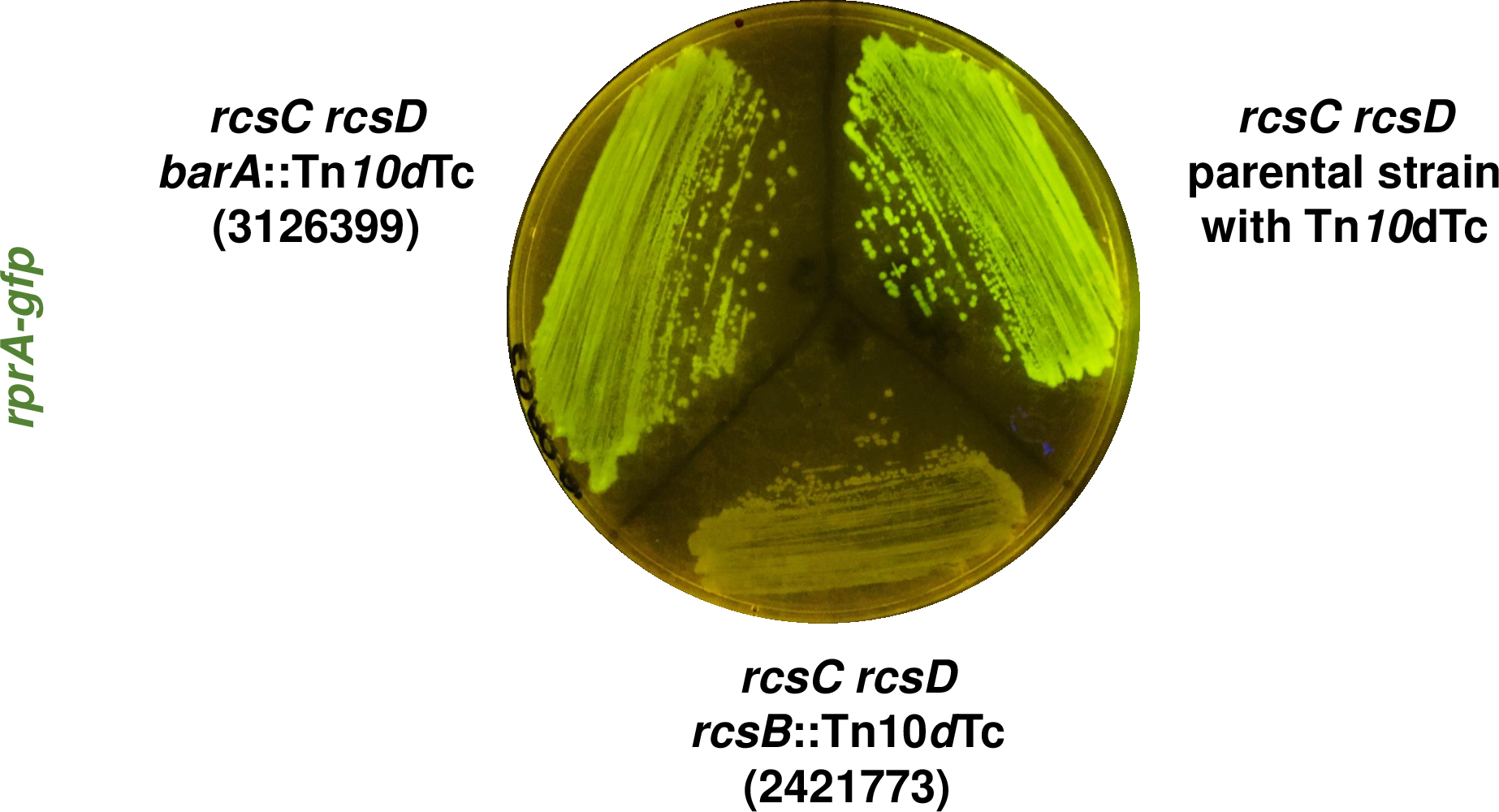

Supplement: S1 Fig — Fluorescence from rcsC rcsD (HS1383) Salmonella harboring plasmid pRprA-GFP (rprA-gfp) and of isogenic mutants with Tn10dTc insertion in the barA and rcsB genes. The genomic location in the Salmonella enterica serovar Typhimurium 14028S genome of each Tn10dTc insertion is indicated below each strain. A derivative of the rcsC rcsD parental strain with a Tn10dTc is also shown. (TIF) [file pgen.1008722.s001.tif]

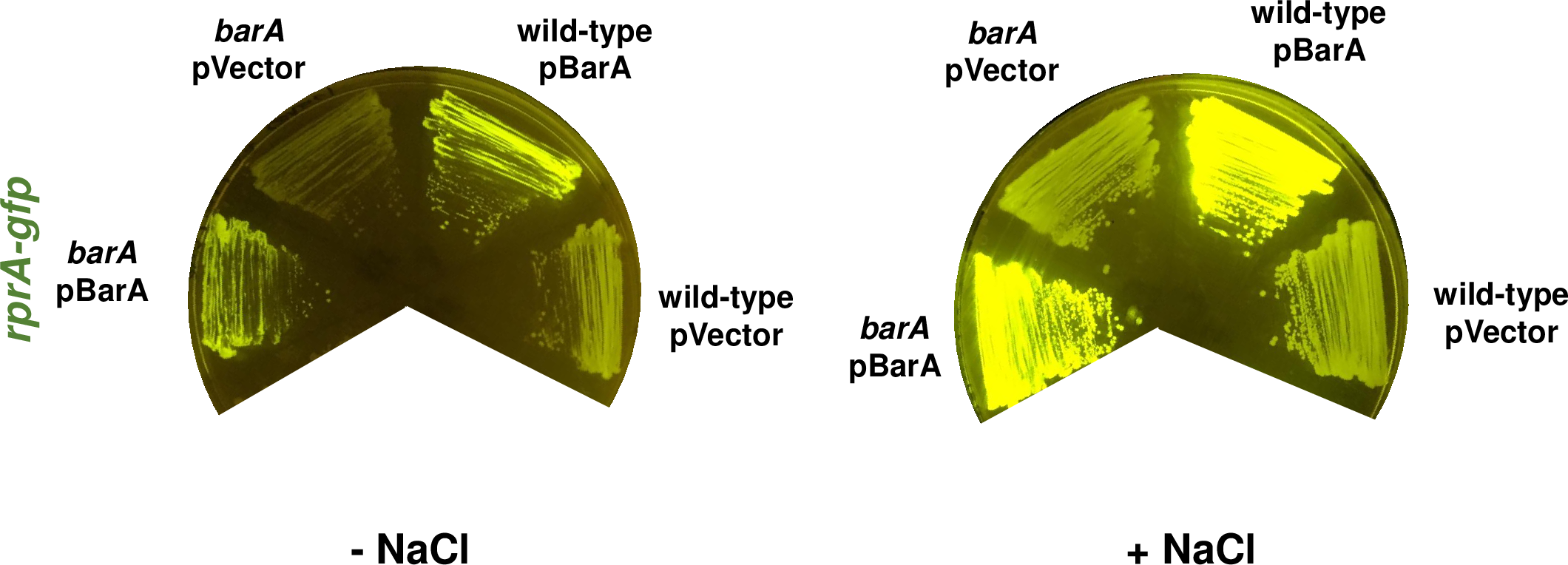

Supplement: S2 Fig — Fluorescence from wild-type (14028s) and HS1520 (barA) Salmonella harboring plasmid pRprA-GFP (rprA-gfp) and pBarA or pVector (empty pACYC184 vector) following 24 h of growth on LB solid medium without (-NaCl) or with (+NaCl) NaCl. Data are representative of two independent experiments, which gave similar results. (TIF) [file pgen.1008722.s002.tif]

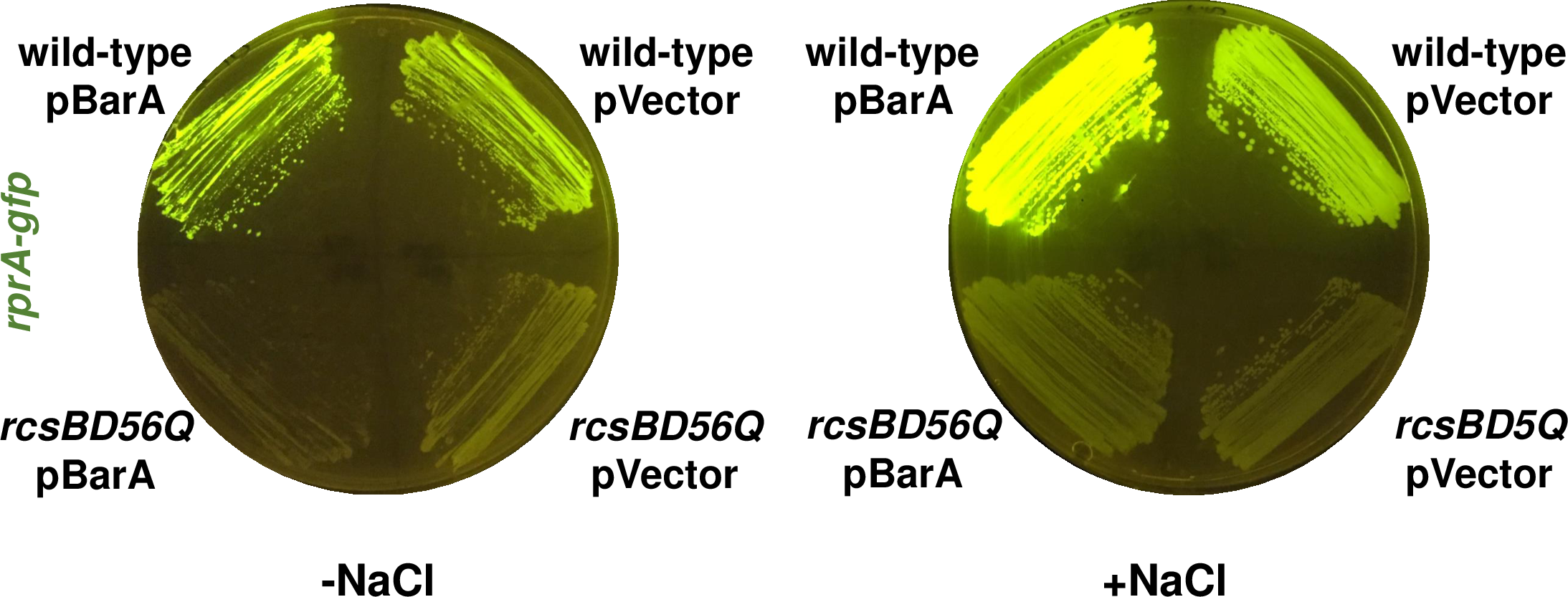

Supplement: S3 Fig — Fluorescence from wild-type (14028s) and rcsBD56Q (HS1483) Salmonella harboring plasmid pRprA-GFP (rprA-gfp) with pBarA or pVector (empty pACYC184 vector) following 24 h of growth on LB solid medium without (-NaCl) or with (+NaCl) NaCl. Data are representative of two independent experiments, which gave similar results. (TIF) [file pgen.1008722.s003.tif]

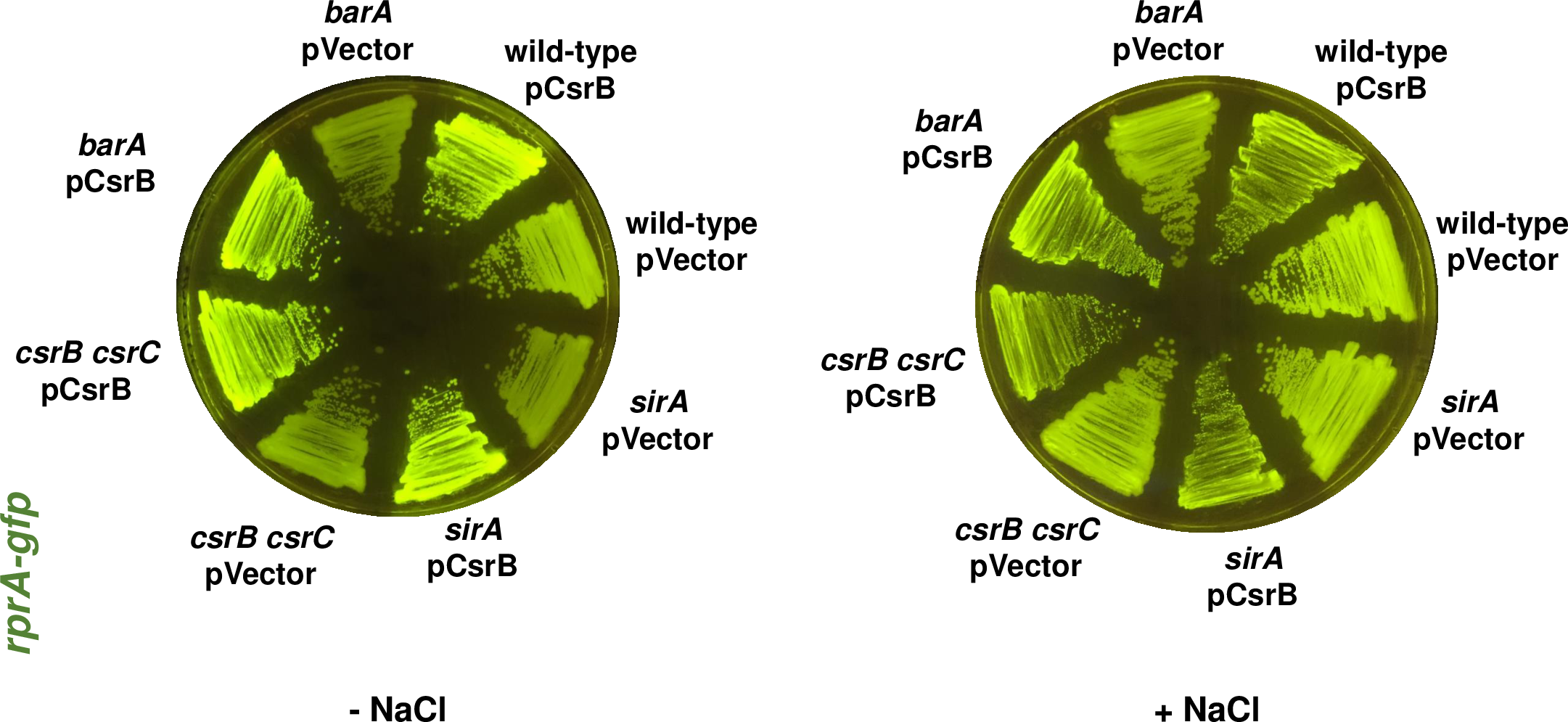

Supplement: S4 Fig — Fluorescence from wild-type (14028s), barA (HS1520), sirA (HS1565) and csrB csrC (HS1651) Salmonella harboring plasmid pRprA-GFP (rprA-gfp) with pCsrB or pVector (empty pACYC184 vector) following 24 h of growth on LB solid medium without (-NaCl) or with (+NaCl) NaCl. Data are representative of two independent experiments, which gave similar results. (TIF) [file pgen.1008722.s004.tif]

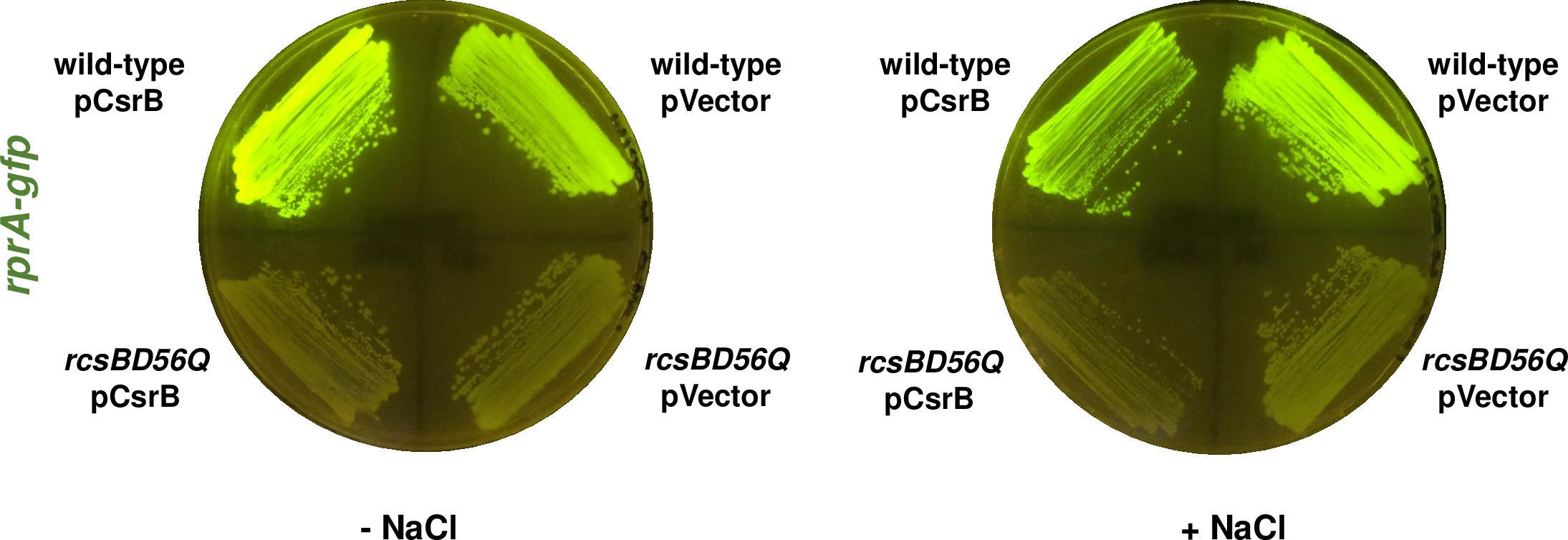

Supplement: S5 Fig — Fluorescence from wild-type (14028s) and rcsBD56Q (HS1483) Salmonella harboring plasmid pRprA-GFP (rprA-gfp) with pCsrB or pVector (empty pACYC184 vector) following 24 h of growth on LB solid medium without (-NaCl) or with (+NaCl) NaCl. Data are representative of two independent experiments, which gave similar results. (TIF) [file pgen.1008722.s005.tif]

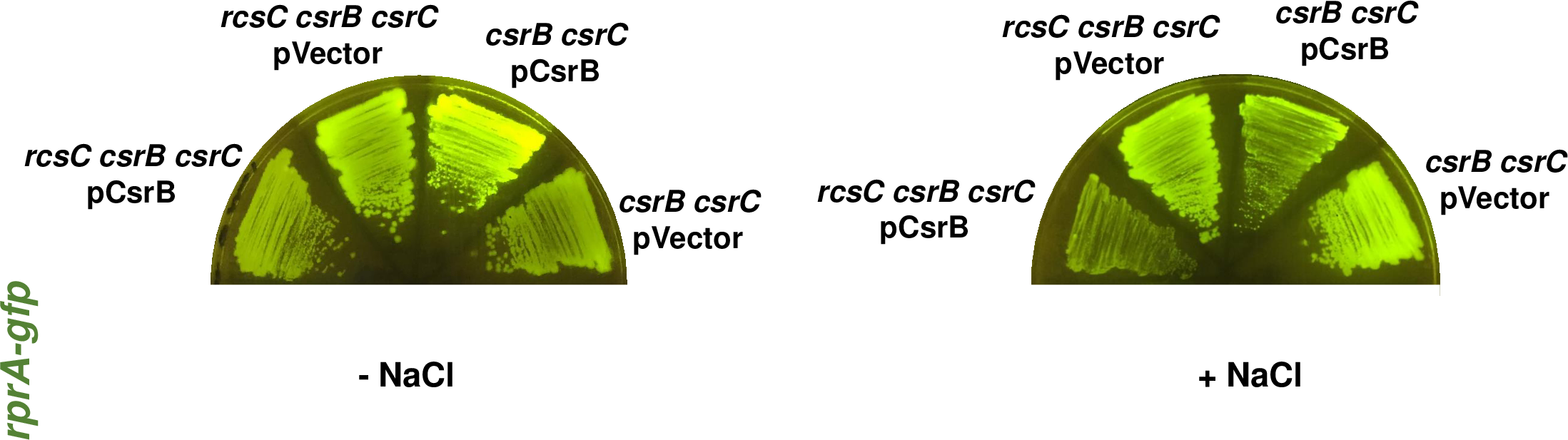

Supplement: S6 Fig — Fluorescence from csrB csrC (HS1651) and csrB csrC rcsC (HS1654), Salmonella harboring plasmid pRprA-GFP (rprA-gfp) with pCsrB or pVector (empty pACYC184 vector) following 24 h of growth on LB solid medium without (-NaCl) or with (+NaCl) NaCl. Data are representative of two independent experiments, which gave similar results. (TIF) [file pgen.1008722.s006.tif]

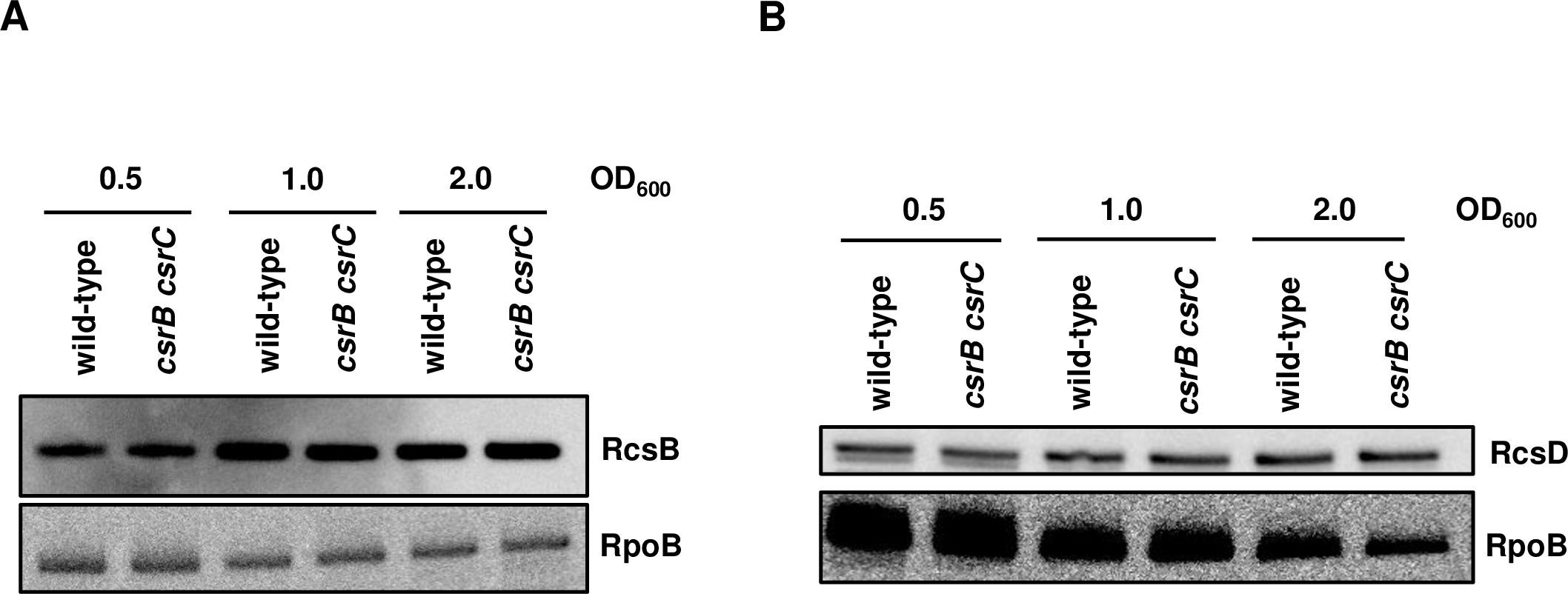

Supplement: S7 Fig — (A) Western blot analysis of crude extracts prepared from rcsB-FLAG (HS717) and rcsB-FLAG csrB csrC (HS1778) Salmonella grown in LB NaCl-free broth. Samples were analyzed with antibodies directed to the FLAG epitope or the RpoB protein. (B) Western blot analysis of crude extracts prepared from rcsD-HA (HS1309) and rcsD-HA csrB csrC (HS2276) Salmonella grown in LB NaCl-free broth. Samples were analyzed with antibodies directed to the HA epitope or the RpoB protein. Data are representative of two independent experiments, which gave similar results. (TIF) [file pgen.1008722.s007.tif]

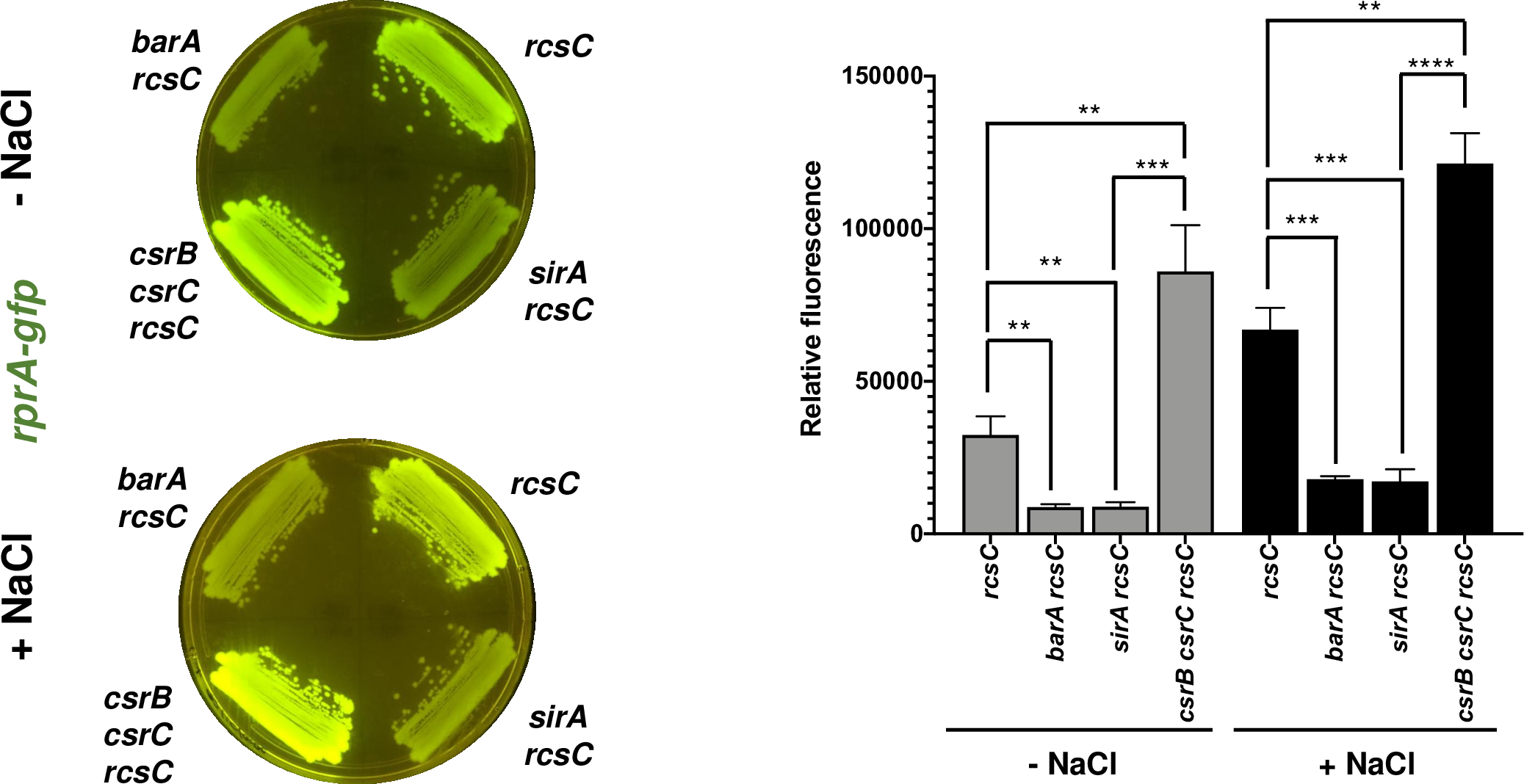

Supplement: S8 Fig — Fluorescence from rcsC (HS1350), barA rcsC (HS1521), sirA rcsC (HS1566) and csrB csrC rcsC (HS1654) Salmonella harboring plasmid pRprA-GFP (rprA-gfp) following 24 h of growth on LB solid medium without (-NaCl) or with (+NaCl) NaCl. Data are representative of three independent experiments, which gave similar results. Quantification of the fluorescence is provided on the right panel of the figure. Values derived from three independent experiments (mean ± standard deviation) were statistically analyzed by Prism 8 using two-tailed unpaired t test. Statistical significance is indicated by *P<0.05, ** P<0.01, *** P<0.001, **** P<0.0001; ns, not significant. Error bars indicate standard deviation. (TIF) [file pgen.1008722.s008.tif]

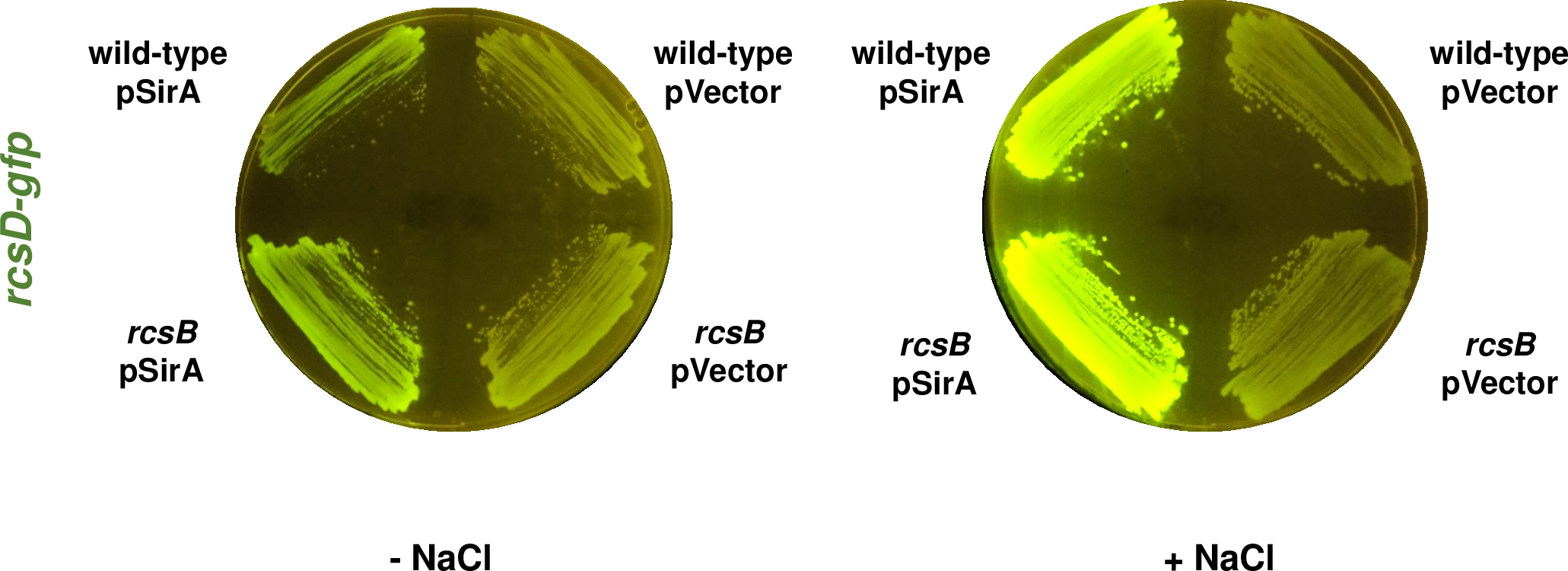

Supplement: S9 Fig — Fluorescence from wild-type (14028s) and rcsB (EG12925) Salmonella harboring plasmid pRprA-GFP with pSirA or pVector (empty pACYC184 vector) following 24 h of growth on LB solid medium without (-NaCl) or with (+NaCl) NaCl. Data are representative of two independent experiments, which gave similar results. (TIF) [file pgen.1008722.s009.tif]

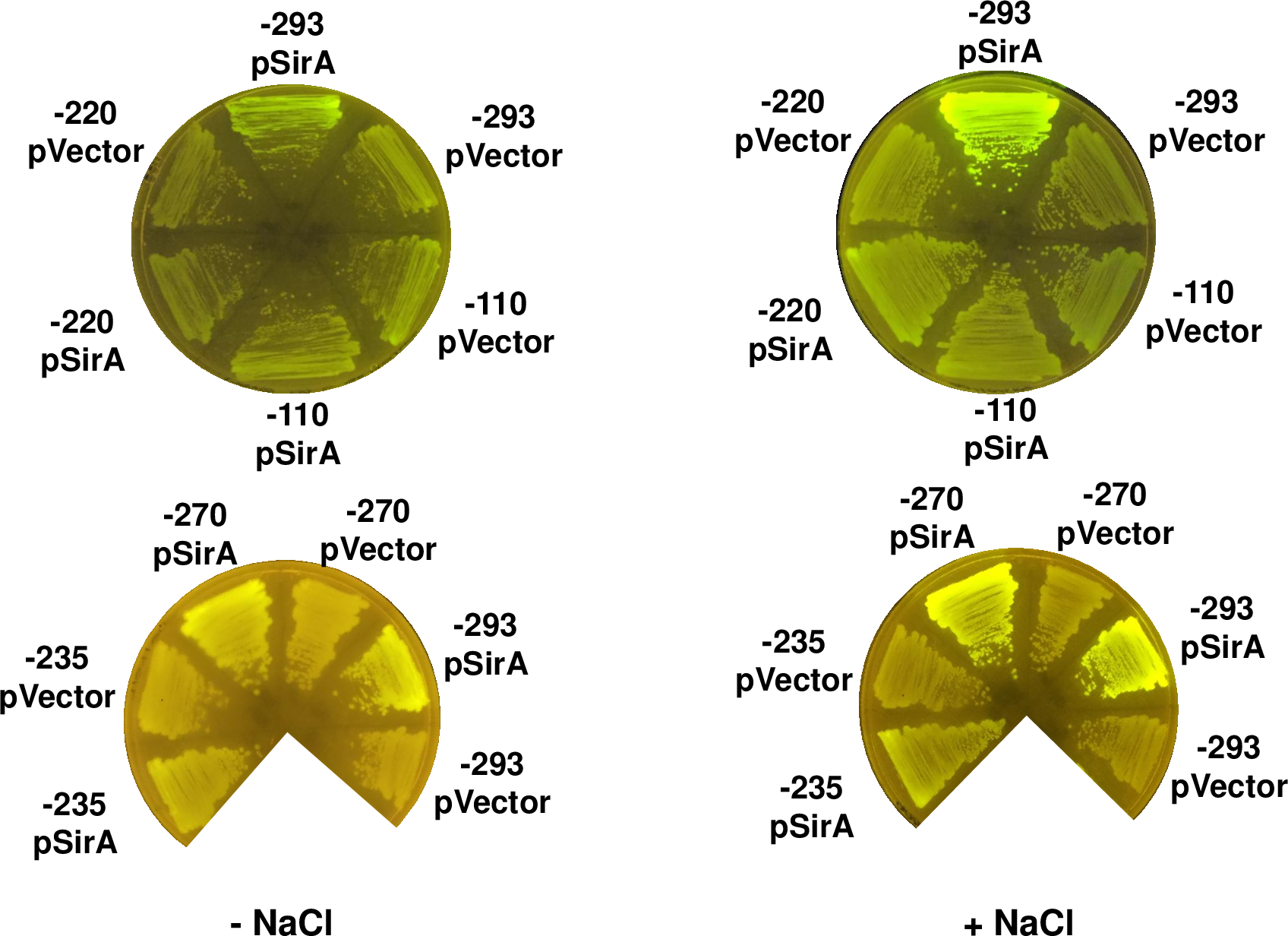

Supplement: S10 Fig — Fluorescence from wild-type (14028s) harboring pRcsD-293-GFP (rcsD-gfp), pRcsD-270-GFP, pRcsD-235-GFP, pRcsD-220-GFP or pRcsD-110-GFP with pSirA or pVector (empty pACYC184 vector) following 24 h of growth on LB solid medium without (-NaCl) or with (+NaCl) NaCl was monitored. The numbers -293, -270, -235, -220 and -110 refer to locations relative to the rcsD start codon. Data are representative of two independent experiments, which gave similar results. (TIF) [file pgen.1008722.s010.tif]

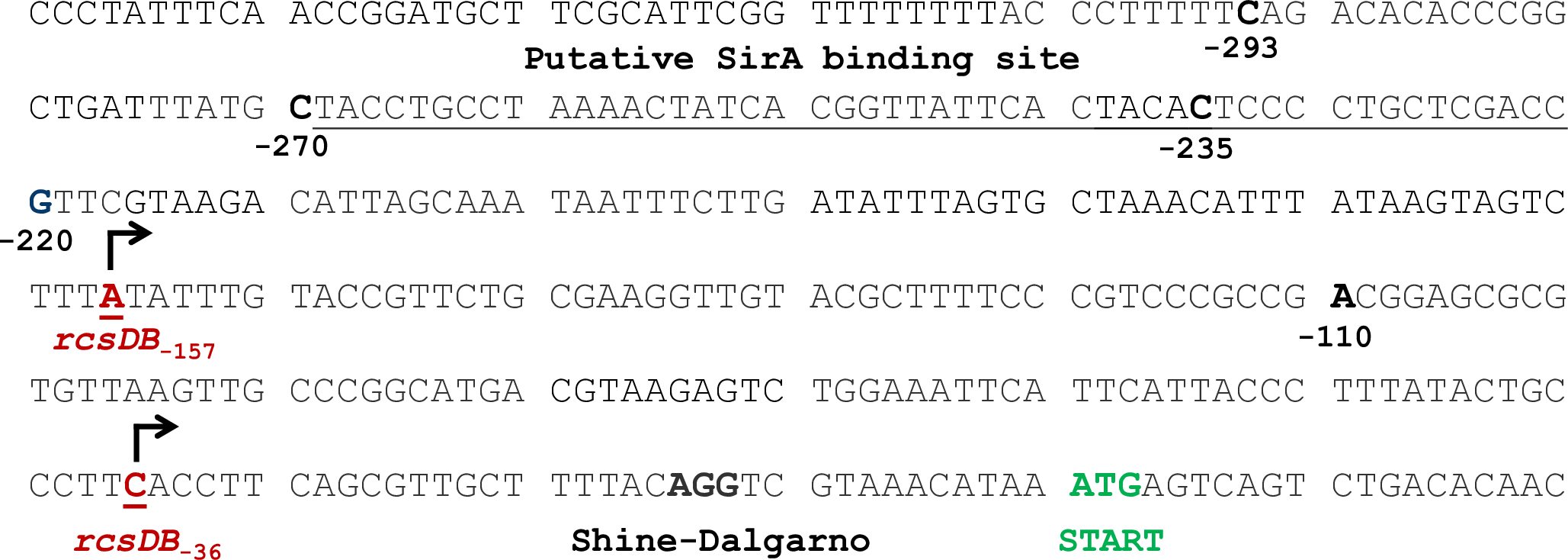

Supplement: S11 Fig — Underlined nucleotides represent the putative SirA binding site based on the results of Fig 6 and S10 Fig. The numbers -293, -270, -235, -220 and -110 refer to locations relative to the rcsD start codon (indicated in bold green letters). (TIF) [file pgen.1008722.s011.tif]

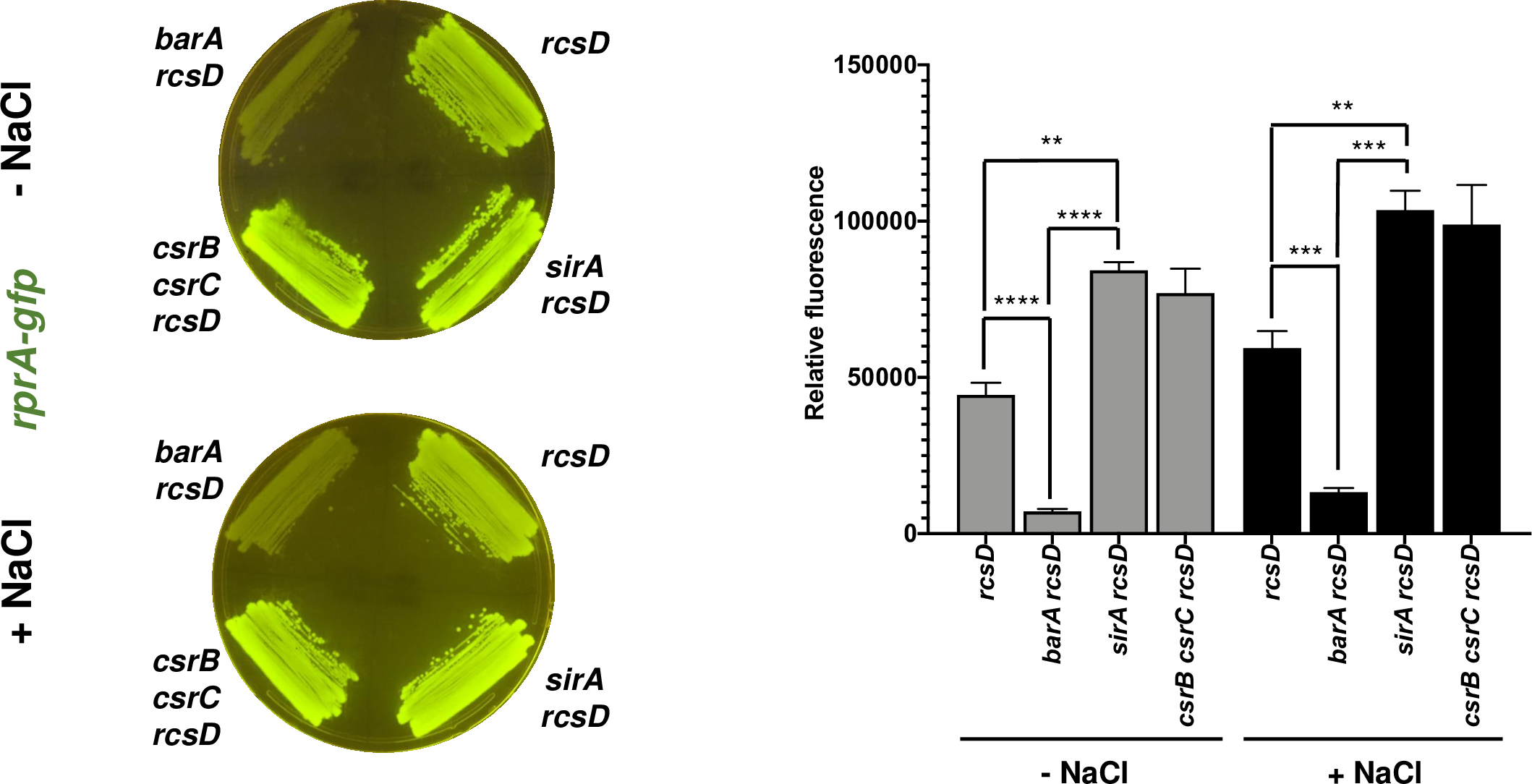

Supplement: S12 Fig — Fluorescence from rcsD (HS1382), barA rcsD (HS1522), sirA rcsD (HS1567) and csrB csrC rcsD (HS1655) Salmonella harboring pRprA-GFP (rprA-gfp) following 24 h of growth on LB solid medium without (-NaCl) or with (+NaCl) NaCl. Data are representative of three independent experiments, which gave similar results. Quantification of the fluorescence is provided on the right panel of the figure. Values derived from three independent experiments (mean ± standard deviation) were statistically analyzed by Prism 8 using two-tailed unpaired t test. Statistical significance is indicated by *P<0.05, ** P<0.01, *** P<0.001; **** P<0.0001; ns, not significant. Error bars indicate standard deviation. (TIF) [file pgen.1008722.s012.tif]

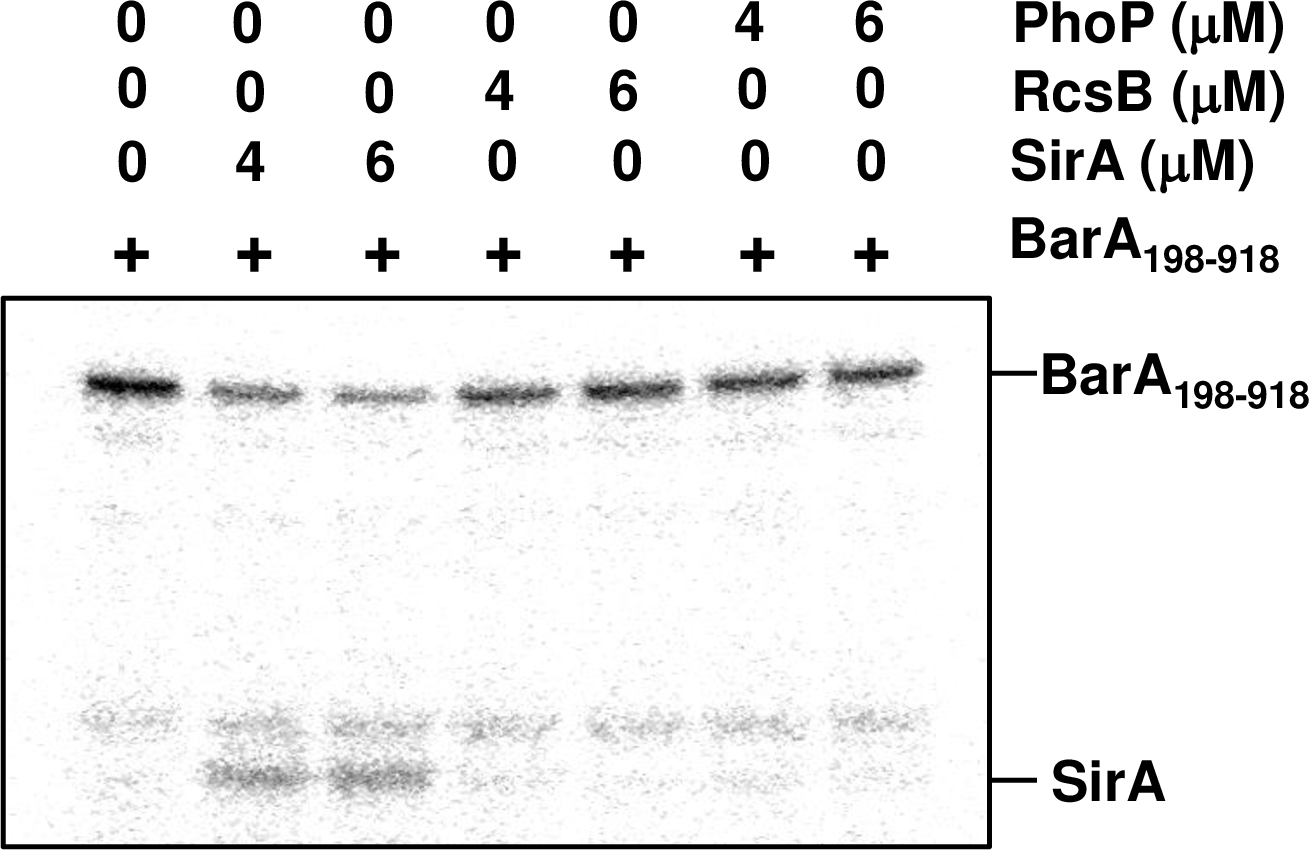

Supplement: S13 Fig — Purified BarA198-918 (0.96 μM final) was first incubated with [γ-32P]ATP for 30 minutes at room temperature. SirA, RcsB or PhoP were then added at the indicated concentrations and the incubation was pursued for 15 min at room temperature before being stopped by the addition of 2X LDS-sample buffer. Data are representative of two independent experiments, which gave similar results. (TIF) [file pgen.1008722.s013.tif]

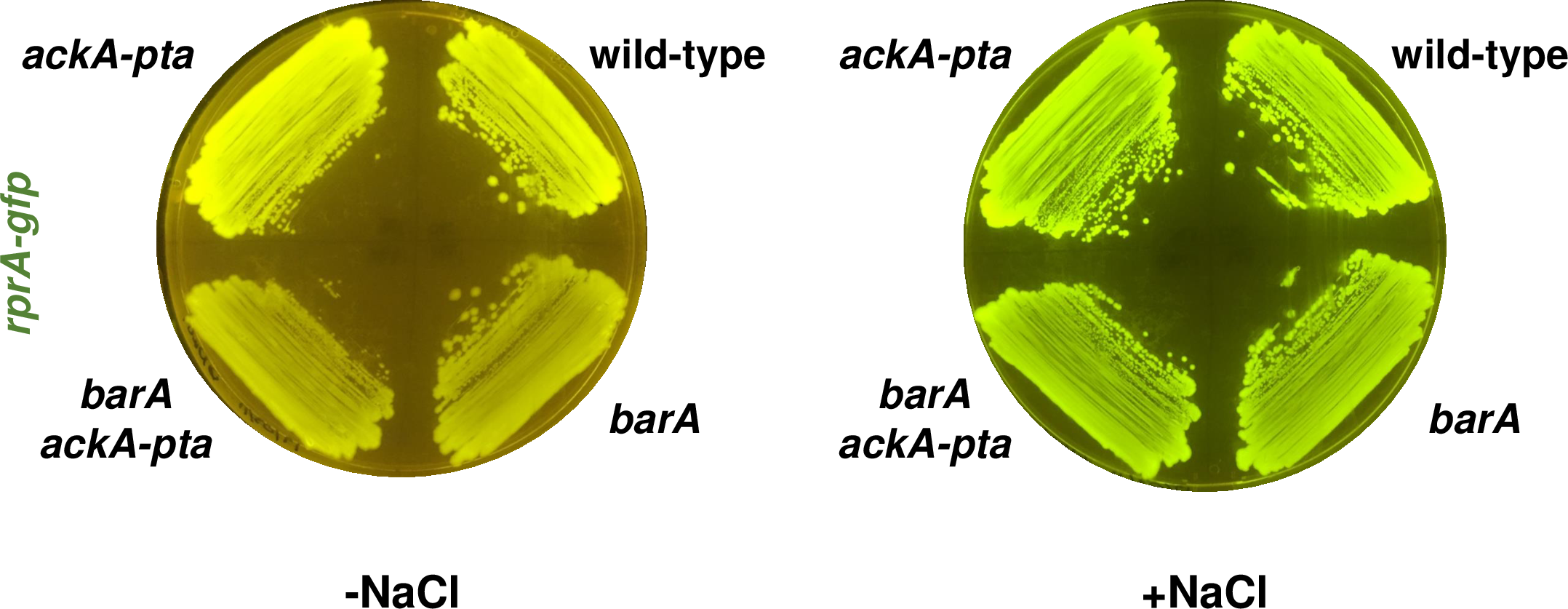

Supplement: S14 Fig — (A) Fluorescence from wild-type (14028s), barA (HS1564), ackA-pta (MP1238) and barA ackA-pta (HS1987) Salmonella harbouring plasmid pRprA-GFP following 24 h of growth on LB solid medium without (-NaCl) or with (+NaCl) NaCl. Data are representative of two independent experiments, which gave similar results. (TIF) [file pgen.1008722.s014.tif]

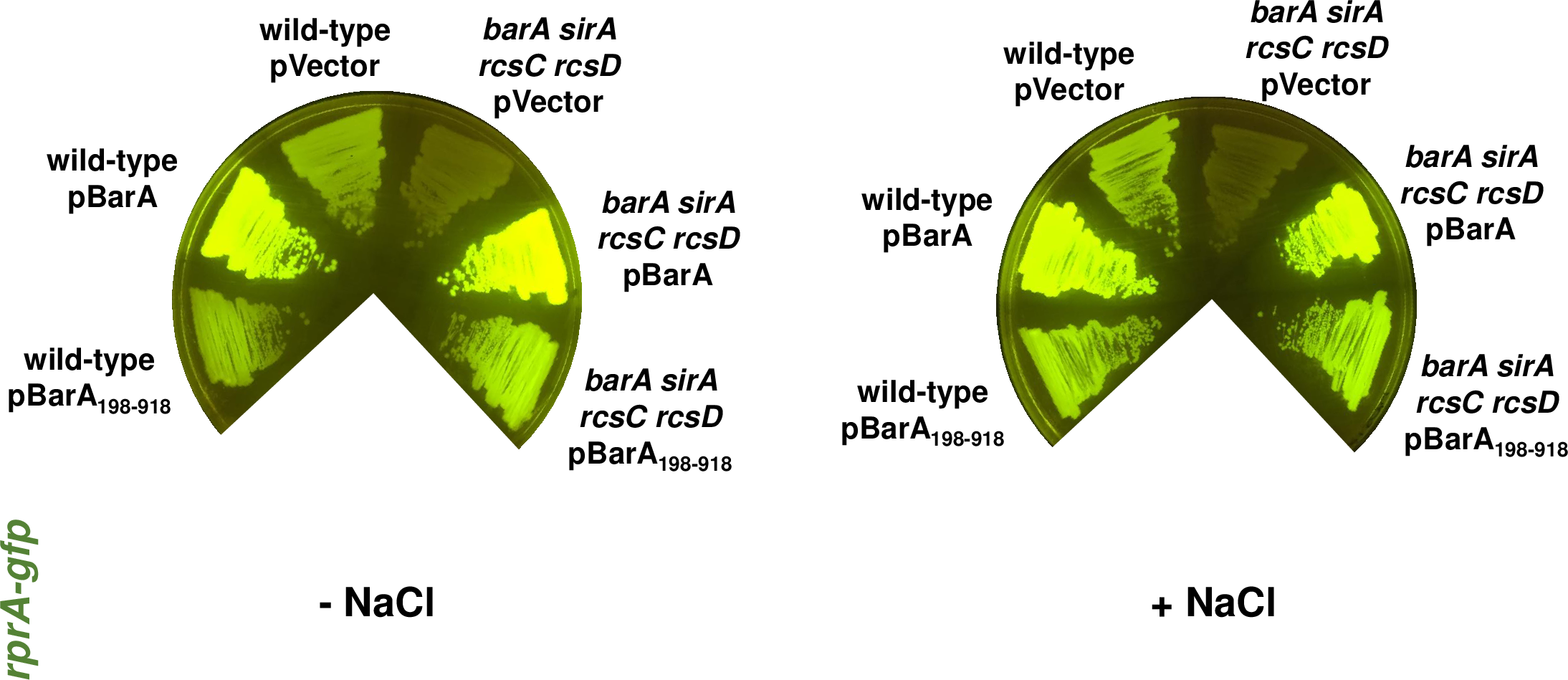

Supplement: S15 Fig — Fluorescence from wild-type (14028) and rcsC rcsD sirA barA (HS1796) Salmonella harboring pRprA-GFP (rprA-gfp) with pSirA or pVector (empty pACYC184 vector) following 24 h of growth on LB solid medium without (-NaCl) or with (+NaCl) NaCl. Data are representative of two independent experiments, which gave similar results. (TIF) [file pgen.1008722.s015.tif]

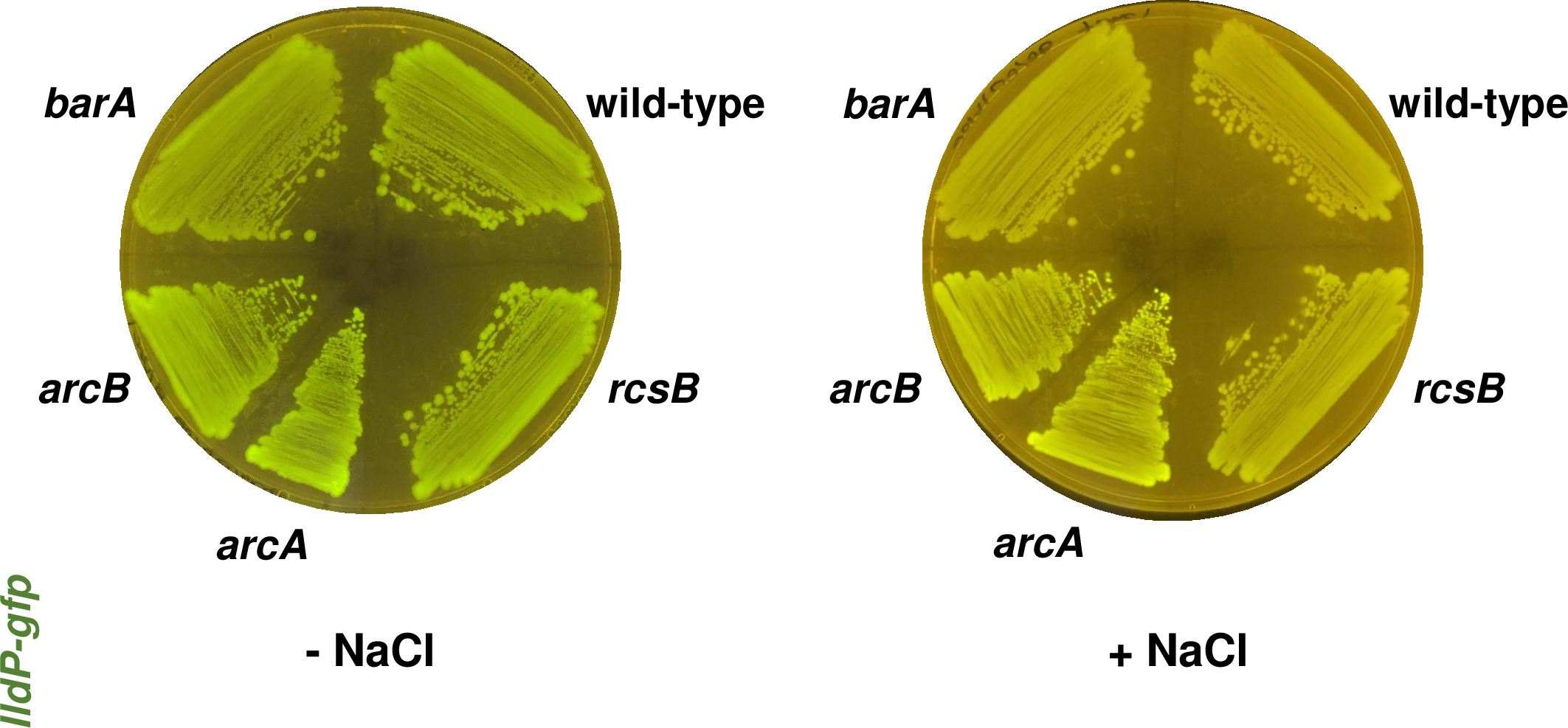

Supplement: S16 Fig — Fluorescence from wild-type (14028s), rcsB (EG12925), barA (HS1520), arcA (MK71) and arcB (EG16900) Salmonella harboring pLldP-GFP (lldP-gfp) following 24 h of growth on LB solid medium without (-NaCl) or with (+NaCl) NaCl. Data are representative of two independent experiments, which gave similar results. (TIF) [file pgen.1008722.s016.tif]

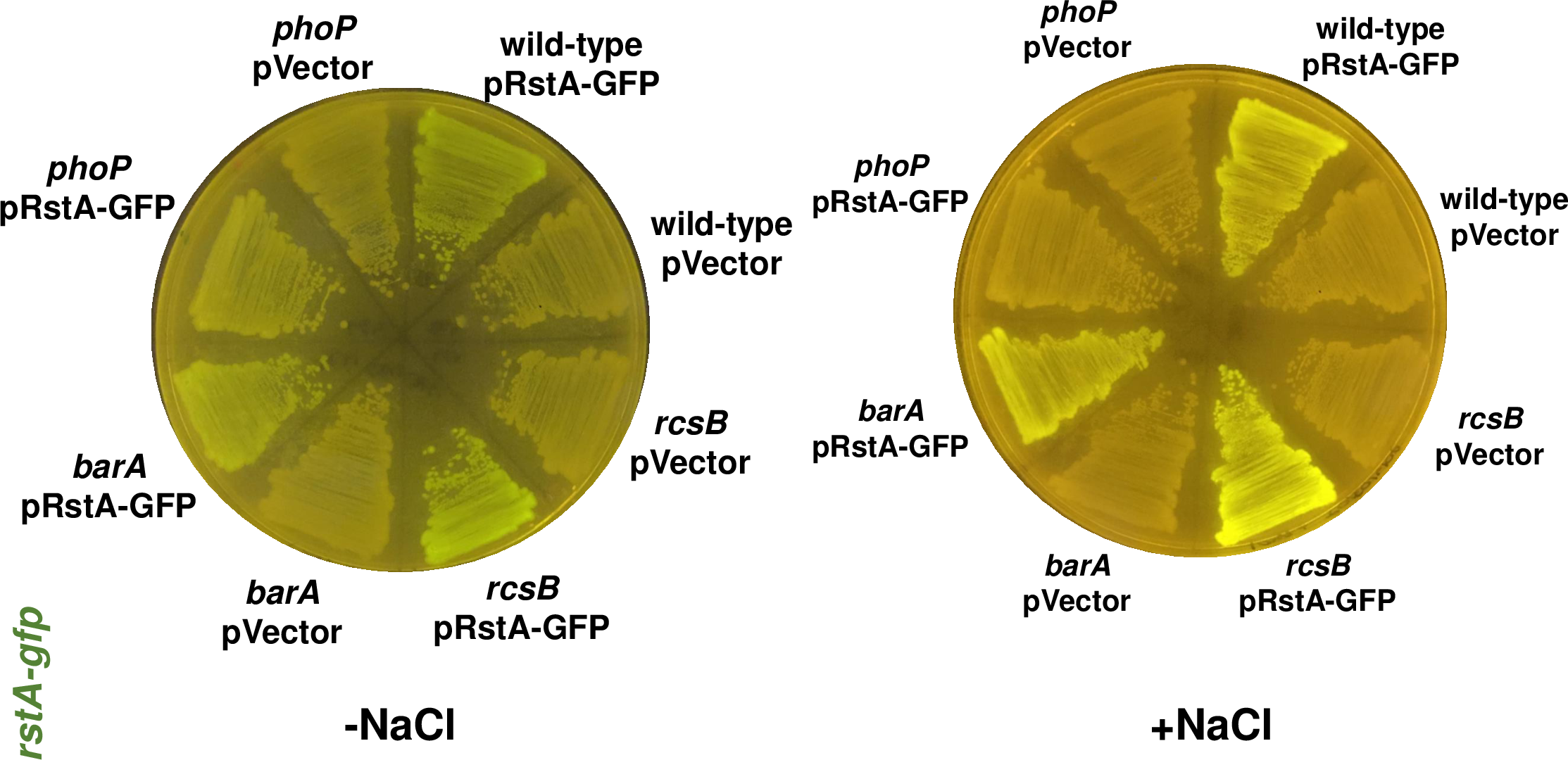

Supplement: S17 Fig — Fluorescence from wild-type (14028s), rcsB (EG12925), barA (HS1520), phoP (MS7953s) Salmonella harboring pRstA-GFP (rstA-gfp) or pVector (empty pMS201) following 24 h of growth on LB solid medium without (-NaCl) or with (+NaCl) NaCl. Data are representative of two independent experiments, which gave similar results. (TIF) [file pgen.1008722.s017.tif]

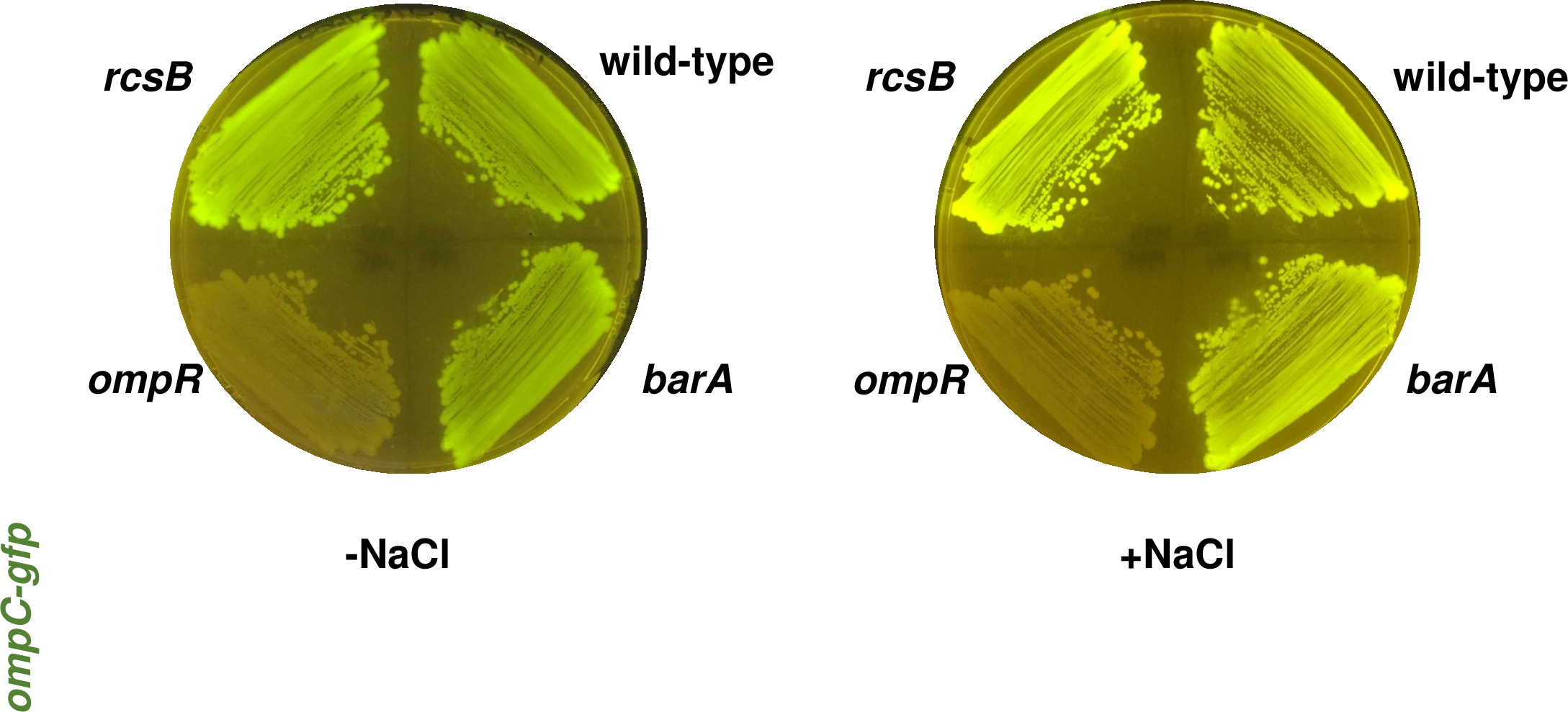

Supplement: S18 Fig — Fluorescence from wild-type (14028s), rcsB (EG12925), barA (HS1520), ompR (EG14379) Salmonella harboring pOmpC-GFP (ompC-gfp) following 24 h of growth on LB solid medium without (-NaCl) or with (+NaCl) NaCl. Data are representative of two independent experiments, which gave similar results. (TIF) [file pgen.1008722.s018.tif]

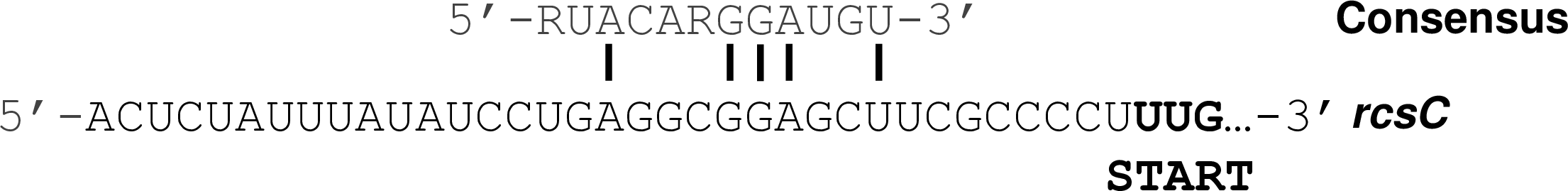

Supplement: S19 Fig — The CsrA binding site consensus sequence is shown above the predicted CsrA binding site in rcsC mRNA. Vertical lines mark the residues in the predicted site that match those in the consensus. (TIF) [file pgen.1008722.s019.tif]
