# Supplementary material for: The phosphorelay BarA/SirA activates the non-cognate regulator RcsB in Salmonella enterica
Source: PLoS Genet. 2020 May 11;16(5):e1008722. doi: 10.1371/journal.pgen.1008722 (PMC7241856; doi:10.1371/journal.pgen.1008722)
Supplement: S1 Table — (DOCX) [file pgen.1008722.s031.docx]

**S1 Table.** Bacterial strains and plasmids used in this study

| **Strains** | **Relevant characteristics** | **Source** |
| --- | --- | --- |
| ***Escherichia coli*** |  |  |
| DH5α | Host strain used for generation and propagation of plasmid constructs | (1) |
| M15 | Expression strain for pQE30 Xa vectors | Qiagen |
| ***Salmonella enterica* serovar Typhimurium** |  |  |
| 14028s | Wild-type | (2) |
| EG12925 | Δ*rcsB* | (3) |
| EG14379 | Δ*ompR*::Cm^R^ | This study, lab stock |
| EG14499 | Δ*rcsF*::Cm^R^ | This study, lab stock |
| EG14873 | *rcsC11* | (4) |
| EG16441 | Δ*barA*::Cm^R^ | This study, lab stock |
| EG16900 | Δ*arcB*::Cm^R^ | This study, lab stock |
| HS539 | *rcsC-3XFLAG*::Cm^R^ | This study |
| HS717 | *rcsB-FLAG*::Cm^R^ | This study |
| HS1309 | *rcsD-HA*:: Cm^R^ | This study |
| HS1326 | Δ*rcsF*::Cm^R^ | This study |
| HS1350 | Δ*rcsC* | This study |
| HS1382 | Δ*rcsD* | This study |
| HS1383 | Δ*rcsC* Δ*rcsD* | This study |
| HS1483 | *rcsBD56Q* | This study |
| HS1520 | Δ*barA*::Cm^R^ | This study |
| HS1521 | Δ*barA*::Cm^R^ Δ*rcsC* | This study |
| HS1522 | Δ*barA*::Cm^R^ Δ*rcsD* | This study |
| HS1523 | Δ*barA*::Cm^R^ Δ*rcsC* Δ*rcsD* | This study |
| HS1564 | Δ*barA* | This study |
| HS1565 | Δ*sirA*::Cm^R^ | This study |
| HS1566 | Δ*sirA*::Cm^R^ Δ*rcsC* | This study |
| HS1567 | Δ*sirA*::Cm^R^ Δ*rcsD* | This study |
| HS1568 | Δ*sirA*::Cm^R^ Δ*rcsC* Δ*rcsD* | This study |
| HS1590 | Δ*barA sirA::*Cm^R^ | This study |
| HS1608 | Δ*csrB*::Cm^R^ | This study |
| HS1609 | Δ*csrC*::Km^R^ | This study |
| HS1651 | Δ*csrB*::Cm^R^ Δ*csrC*::Km^R^ | This study |
| HS1654 | Δ*csrB*::Cm^R^ Δ*csrC*::Km^R^ Δ*rcsC* | This study |
| HS1655 | Δ*csrB*::Cm^R^ Δ*csrC*::Km^R^ Δ*rcsD* | This study |
| HS1656 | Δ*csrB*::Cm^R^ Δ*csrC*::Km^R^ Δ*rcsC* Δ*rcsD* | This study |
| HS1722 | Δ*csrB* Δ*csrC* | This study |
| HS1774 | Δ*barA* Δ*rcsC* Δ*rcsD* | This study |
| HS1778 | *rcsB-FLAG*::Cm^R^ Δ*csrB* Δ*csrC* | This study |
| HS1796 | Δ*barA* Δ*rcsC* Δ*rcsD sirA*::Cm | This study |
| HS1987 | Δ*barA* Δ*ackA-pta*::Cm^R^ | This study |
| HS2263 | *rcsC-3XFLAG*::Cm^R^ Δ*csrB* Δ*csrC* | This study |
| HS2276 | *rcsD-HA*:: Cm^R^ Δ*csrB* Δ*csrC* | This study |
| MK71 | Δ*arcA*::Cm^R^ | This study, lab stock |
| MP1238 | Δ*ackA-pta*::Cm^R^ | This study, lab stock |
| MS7953s | *phoP7953*::Tn*10* | (2) |
| TH338 | F::Tn*10*dTc | K. Hugues |
| **Plasmids** | **Relevant characteristics** | **Source** |
| pCP20 | rep_pSC101_^ts^ λ cI857 FLP Amp^R^ Cm^R^ | (5) |
| pKD3 | rep_R6Kg_ Amp^R^ FRT Cm^R^ FRT | (5) |
| pKD4 | rep_R6Kg_ Amp^R^ FRT Km^R^ FRT | (5) |
| pKD46 | rep_pSC101_^ts^ Amp^R^ P_araBAD_-γβexo | (5) |
| pFPV25 | ori_pMB1_, Ap^R^, promoterless gfp | (6) |
| pACYC184 | rep_p15A_ Cm^R^ Tet^R^ | (7) |
| pNK972 | P_tac_‐tnpA (Amp^R^) | (8) |
| pREP4 | *lacI*, Km^R^ | Qiagen |
| pLldP-GFP | rep_pMB1_ Amp^R^ P*_lldP_*-*gfp* | This study |
| pOmpC-GFP | rep_pMB1_ Amp^R^ P*_ompC_*-*gfp* | This study |
| pRcsD_-293_-GFP | rep_pMB1_ Amp^R^ P*_rcsD-293_*-*gfp* | This study |
| pRcsD_-270_-GFP | rep_pMB1_ Amp^R^ P*_rcsD-270_*-*gfp* | This study |
| pRcsD_-235_-GFP | rep_pMB1_ Amp^R^ P*_rcsD-235_*-*gfp* | This study |
| pRcsD_-220_-GFP | rep_pMB1_ Amp^R^ P*_rcsD-220_*-*gfp* | This study |
| pRcsD_-110_-GFP | rep_pMB1_ Amp^R^ P*_rcsD-110_*-*gfp* | This study |
| pRprA-GFP | rep_pMB1_ Amp^R^ P*_rprA_*-*gfp* | This study |
| pBarA | pACYC184-*barA* (Tet^R^) | This study |
| pSirA | pACYC184-*sirA* (Tet^R^) | This study |
| pSLC-242 | rep_R6Kγ_ Amp^R^ FRT-(Cm^R^ P*_rhaB_*-*relE*)-FRT | (9) |
| pRstA-GFP (pMS201-*rstA4*) | rep_pSC101_ Km^R^ p*_rstA_* *gfp* | (10) |
| pQE30 Xa | Expression vector for His-tagged proteins Amp^R^ | Qiagen |
| pQE30-BarA_198-918_ | pQE30 Xa vector carrying His6-*barA*_198-918_ (*barA* coding region; amino acids 198 to 918). Amp^R^ | This study |
| pQE30-SirA | pQE30 Xa vector carrying His6-*sirA*. Amp^R^ | This study |
| pQE30-RcsB | pQE30 Xa vector carrying His6-*rcsB*. Amp^R^ | This study |
| pQE30-PhoP | pQE30 Xa vector carrying His6-*phoP*. Amp^R^ | This study |

**References**

1. D. Hanahan, Studies on transformation of Escherichia coli with plasmids. *J Mol Biol* **166**, 557-580 (1983).

2. P. I. Fields, R. V. Swanson, C. G. Haidaris, F. Heffron, Mutants of Salmonella typhimurium that cannot survive within the macrophage are avirulent. *Proc Natl Acad Sci U S A* **83**, 5189-5193 (1986).

3. C. Mouslim, E. A. Groisman, Control of the Salmonella ugd gene by three two-component regulatory systems. *Mol Microbiol* **47**, 335-344 (2003).

4. C. Mouslim, M. Delgado, E. A. Groisman, Activation of the RcsC/YojN/RcsB phosphorelay system attenuates Salmonella virulence. *Mol Microbiol* **54**, 386-395 (2004).

5. K. A. Datsenko, B. L. Wanner, One-step inactivation of chromosomal genes in Escherichia coli K-12 using PCR products. *Proc Natl Acad Sci U S A* **97**, 6640-6645 (2000).

6. R. H. Valdivia, S. Falkow, Bacterial genetics by flow cytometry: rapid isolation of Salmonella typhimurium acid-inducible promoters by differential fluorescence induction. *Mol Microbiol* **22**, 367-378 (1996).

7. A. C. Chang, S. N. Cohen, Construction and characterization of amplifiable multicopy DNA cloning vehicles derived from the P15A cryptic miniplasmid. *J Bacteriol* **134**, 1141-1156 (1978).

8. J. C. Way, M. A. Davis, D. Morisato, D. E. Roberts, N. Kleckner, New Tn10 derivatives for transposon mutagenesis and for construction of lacZ operon fusions by transposition. *Gene* **32**, 369-379 (1984).

9. V. Khetrapal *et al.*, A set of powerful negative selection systems for unmodified Enterobacteriaceae. *Nucleic Acids Res* **43**, e83 (2015).

10. I. Zwir, T. Latifi, J. C. Perez, H. Huang, E. A. Groisman, The promoter architectural landscape of the Salmonella PhoP regulon. *Mol Microbiol* **84**, 463-485 (2012).
