# Supplementary material for: The phosphorelay BarA/SirA activates the non-cognate regulator RcsB in Salmonella enterica
Source: PLoS Genet. 2020 May 11;16(5):e1008722. doi: 10.1371/journal.pgen.1008722 (PMC7241856; doi:10.1371/journal.pgen.1008722)
Supplement: S2 Table — (DOCX) [file pgen.1008722.s032.docx]

**S2 Table.** Oligonucleotides sequences used in this study

| **Name** | **Sequence (5′ -> 3′)** | **Purpose** | **Source** |
| --- | --- | --- | --- |
| W804 | GTTATTGTCTCATGAGCGGA | Sequencing of pFPV25 inserts | This study |
| W2504 | GCTGGCGGTGTATGCCGAGCGGGTACGTAAAACGCGGGCAGACTACAAGGACCACGACGGTGACTACAAGGACCACGACATCGACTACAAGGACGACGACGACAAGTGACATATGAATATCCTCCTTA | Generation of *rcsC-3XFLAG*::Cm^R^ strain | This study |
| W2505 | TTTTACAGGCCGGACAGGCGACGCCGCCATCCGGCATTTTGTGTAGGCTGGAGCTGCTTC | Generation of *rcsC-3XFLAG*::Cm^R^ strain | This study |
| W2506 | CAGCGTATCCGTCAGCTTG | *rcsC-3XFLAG*::Cm^R^ verification | This study |
| W2507 | GAATGATATCGCGCTGCTCAACT | *rcsC-3XFLAG*::Cm^R^ verification | This study |
| W2541 | ATAAGCGTAGCGCCATCAGGCTGGGTAACATAAAAGCGATGTGTAGGCTGGAGCTGCTTC | Generation of *rcsB-FLAG*::Cm^R^ strain | This study |
| W2542 | ATCGCCAAGAAGCTCAAC | *rcsB-FLAG*::Cm^R^ verification | This study |
| W2543 | GAGCGGGTACGTAAAACGC | *rcsB-FLAG*::Cm^R^ verification | This study |
| W2585 | AATAATTACGTTCATATTGTTCATGTATTGGGCTACCTTGGTGTAGGCTGGAGCTGCTTC | Generation of *rcsD-HA*::Cm^R^ strain | This study |
| W2586 | TGCCATGCTTAATCTGGTAC | *rcsD-HA*::Cm^R^ verification | This study |
| W2587 | CAACATTCACCCACTCGATT | *rcsD-HA*::Cm^R^ verification | This study |
| W2895 | CTATCTCTCTTCCGTCACCCTGAGTCCGACAGACAAAGAAGACTACAAGGACGACGATGACAAGTAACATATGAATATCCTCCTTA | Generation of *rcsB-FLAG*::Cm^R^ strain | This study |
| W3102 | CAGTTGAATTCCAGACACACCCGGCTGATTTATG | Cloning of *rcsD* promoter region (from position -293 relative to *rcsD* ATG). | This study |
| W3103 | GCATCGGATCCGACCGTTGTGTCAGACTGACTCAT | Cloning of *rcsD* promoter region | This study |
| W3228 | GCTCGACCGTTCGTAAGACATTAG | DNA template generation for *rcsD* primer extension sequencing ladder | This study |
| W3229 | GAGAATCGGGTAGAGAC | DNA template generation for *rcsD* primer extension sequencing ladder | This study |
| W3246 | ACCGTCATTCACTTCTGAATG | Δ*rcsD*::Km^R^ verification | This study |
| W3247 | CTCGTTTAACGTTACACCT | Δ*rcsD*::Km^R^ verification | This study |
| W3522 | AGCGATGACGACGTTG | Δ*rcsC*::Cm^R^ verification | This study |
| W3523 | GAAACTGGGCGTAGAGA | Δ*rcsC*::Cm^R^ verification | This study |
| W3556 | CGTCATTTACCGCTACCTTAGTCACACTCTATTTACATCCgtgtaggctggagctgcttc | *rcsC* inactivation | This study |
| W3557 | AACGTTGTTTCTCTTCCGCCAGCGCGTTCGCCGTCACGCCcatatgaatatcctccttag | *rcsC* inactivation | This study |
| W4171 | CAGCACAATGATCAGCAATAAG | *rcsD* primer extension | This study |
| W4246 | ATATATCAGCGACATTGACGCCTACGTCAAAAGCTTGCTGTACCCATACGATGTTCCAGATTACGCTTAGCATATGAATATCCTCCTTA | Generation of *rcsD-HA*::Cm^R^ strain | This study |
| W4304 | CAGTTGAATTCCGATGCTTTAGCGACGTTTATC | Cloning of *rprA* promoter region | This study |
| W4305 | GCATCGGATCCGATTTATAACCGTGTGCTAATAGTAG | Cloning of *rprA* promoter region | This study |
| W4311 | CCTTCACCTTCAGCGTTGCTTTTACAGGTCGTAAACATAAgtgtaggctggagctgcttc | *rcsD* inactivation | This study |
| W4312 | CGTCACATCGACCATCACGCTCACGTCGTCCAGCAGGCGCcatatgaatatcctccttag | *rcsD* inactivation | This study |
| W4387 | TTGATCAACAACCTGCCGAAATTAGATGCGCATGTGTTGAgtgtaggctggagctgcttc | Generation of *rcsBD56Q* strain | This study |
| W4388 | TGTACTTGATCAAGGTGATCCCATCGCCGTATTTATCTCCcatatgaatatcctccttag | Generation of *rcsBD56Q* strain | This study |
| W4389 | GACCACCCGATTGTACTG | Sequencing primer for *rcsBD56Q* allele insertion confirmation | This study |
| W4390 | GTTGTTCATGGTCAGAACGA | Sequencing primer for *rcsBD56Q* allele insertion confirmation | This study |
| W4391 | TTGATCAACAACCTGCCGAAATTAGATGCGCATGTGTTGATCACTCAGCTCTCCATGCCGGGAGATAAATACGGCGATGGGATCACCTTGATCAAGTACA | Generation of *rcsBD56Q* strain | This study |
| W4392 | TGTACTTGATCAAGGTGATCCCATCGCCGTATTTATCTCCCGGCATGGAGAGCTGAGTGATCAACACATGCGCATCTAATTTCGGCAGGTTGTTGATCAA | Generation of *rcsBD56Q* strain. | This study |
| W4448 | GCCTTCTTATTCGGCCTTGAATTGATCATATGCGG | Identification of Tn*10*dTn insertion location | This study |
| W4449 | GGCCACGCGTCGACTAGTACNNNNNNNNNNGATAT | Identification of Tn*10*dTn insertion location | This study |
| W4451 | CTTTTTCCGTGATGGTAACC | Identification of Tn*10*dTn insertion location | This study |
| W4452 | GGCCACGCGTCGACTAGTAC | Identification of Tn*10*dTn insertion location | This study |
| W4470 | TAACTATCAGTAGCGTTATCCCTATTCTGGAGATATTCCTcatatgaatatcctccttag | *sirA* inactivation | This study |
| W4471 | CATCAGCATAATCTGCAACTCGCGTTCAGACAAACTGGCGgtgtaggctggagctgcttc | *sirA* inactivation | This study |
| W4472 | CGAGAGCAAAATCGAATACC | Δ*sirA*::Cm^R^ verification | This study |
| W4473 | CTGAACATACGATAGCGAT | Δ*sirA*::Cm^R^ verification | This study |
| W4541 | AAGACAAGGTGAAACAGGCGATTCTATCTTCGTCGACAGGcatatgaatatcctccttag | *csrB* inactivation | This study |
| W4542 | GTCATGTTAAAAACCTCAATGATGAAAATCTGGCGCGAAGgtgtaggctggagctgcttc | *csrB* inactivation | This study |
| W4543 | GATATCAGCGGATACTGAG | Δ*csrB*::Cm^R^ verification | This study |
| W4544 | CAGCAGCGTTTCAAGCGTA | Δ*csrB*::Cm^R^ verification | This study |
| W4545 | GTATCTTGTGAGTTTACCCCAAAAGAGTAAAGTAATGCACgtgtaggctggagctgcttc | *csrC* inactivation | This study |
| W4546 | TCAGTATAGAATTGAGGCGGAATCTAGCAGAAAGCAAGCAcatatgaatatcctccttag | *csrC* inactivation | This study |
| W4547 | TGCGACATGACAGAGTCGTTG | Δ*csrC*::Km^R^ verification | This study |
| W4548 | CTTCACGCGTTTTACGACGC | Δ*csrC*::Km^R^ verification | This study |
| W4587 | CAGTTGAATTCGAAACGCAGCGTCGAAGAAGCAAACAC | Cloning of *lldP* promoter region | This study |
| W4588 | GCATCGGATCCGGATAATGACAACAAATAGTTAAC | Cloning of *lldP* promoter region | This study |
| W4592 | CAGTTGAATTCCGAATATCAGCCTAATATCTTCTTCG | Cloning of *barA* ORF | This study |
| W4593 | CAGTTGAATTCCTATTCTGGAGATATTCCTTTGATCAACGTTC | Cloning of *sirA* ORF | This study |
| W4595 | CAGTTGAATTCCAAATATTTCACTCACTGGCTTGTTAACG | Cloning of *sirA* ORF | This study |
| W4596 | AGACCGTTCAGCTGGATATTAC | Sequencing of pACYC184 inserts | This study |
| W4597 | CATATCACCAGCTCACCGTC | Sequencing of pACYC184 inserts | This study |
| W4598 | GCGAATTAATACGACTCACTATAGGGCTTAAGTATAAGGAGGAAAAAATATGACCAACTACAGCCTGCGCGCACGCATG | Generation of *barA-HA* template | This study |
| W4599 | AAACCCCTCCGTTTAGAGAGGGGTTATGCTAGTCAAGCGTAATCTGGAACATCGTATGGGTAGCCTAATATCTTCTTCGCTTCACG | Generation of *barA-HA* template | This study |
| W4608 | GCGAATTAATACGACTCACTATAGGGCTTAAGTATAAGGAGGAAAAAATATGAACAATATGAACGTAATTATTGCCGATG | Generation of *rcsB-FLAG* template | This study |
| W4609 | AAACCCCTCCGTTTAGAGAGGGGTTATGCTAGTTACTTGTCATCGTCGTCCTTGTAGTCTTCTTTGTCTGTCGGACTCAGGGTGACGGAAG | Generation of *rcsB-FLAG* template | This study |
| W4621 | GCGAATTAATACGACTCACTATAGGGCTTAAGTATAAGGAGGAAAAAATATGATGCGCGTACTGGTTGTAGAGGATAATGC | Generation of *phoP-FLAG* template | This study |
| W4622 | AAACCCCTCCGTTTAGAGAGGGGTTATGCTAGTTACTTGTCATCGTCGTCCTTGTAGTCGCGCAATTCAAAAAGATATCCTTGTCCGCGTAC | Generation of *phoP-FLAG* template | This study |
| W4623 | GCGAATTAATACGACTCACTATAGGGCTTAAGTATAAGGAGGAAAAAATATGATCAACGTTCTTCTTGTTGATGACCACGAACTGGTG | Generation of *sirA-FLAG* template | This study |
| W4624 | AAACCCCTCCGTTTAGAGAGGGGTTATGCTAGTCACTTGTCATCGTCGTCCTTGTAGTCCTGGCTTGTTAACGTCTCCGCATTACACAGGCCA | Generation of *sirA-FLAG* template | This study |
| W4640 | CAGTTGAATTCGTCCCATAACGGAACTCCATGACCAACTACAGCCTGCGCGCACGCATGATG | Cloning of *barA* ORF | This study |
| W4703 | CCCGGATCCCTTATGCGCGATGTCACCGGGCCTATCCG | Cloning of *barA*_198-918_ into pQE30 Xa vector | This study |
| W4704 | CCCGGATCCTCAGCCTAATATCTTCTTCGCTTCACG | Cloning of *barA*_198-918_ into pQE30 Xa vector | This study |
| W4705 | CCCGGATCCAACAATATGAACGTAATTATTGCCGATG | Cloning of *rcsB* into pQE30 Xa vector | This study |
| W4706 | CCCGGATCCTTATTCTTTGTCTGTCGGACTCAG | Cloning of *rcsB* into pQE30 Xa vector | This study |
| W4707 | CCCGGATCCATCAACGTTCTTCTTGTTGATGACCAC | Cloning of *sirA* into pQE30 Xa vector | This study |
| W4708 | CCCGGATCCTCACTGGCTTGTTAACGTCTCCGCAT | Cloning of *sirA* into pQE30 Xa vector | This study |
| W4709 | CCCGGATCCCGCGTACTGGTTGTAGAGGATAATGC | Cloning of *phoP* into pQE30 Xa vector | This study |
| W4710 | CCCGGATCCTTAGCGCAATTCAAAAAGATATCCTTGTC | Cloning of *phoP* into pQE30 Xa vector | This study |
| W4711 | CGGATAACAATTTCACACAG | Sequencing of pQE30 Xa inserts | This study |
| W4717 | CAGTTGAATTCGTCGACAGGGAGTCGTACAACG | Cloning of CsrB | This study |
| W4718 | CAGTTGAATTCATGAGTCGTCATGTTAAAAACCTCAATGA | Cloning of CsrB | This study |
| W4725 | CAGTTGAATTCGTTCGTAAGACATTAGCAAATAATTTCTTG | Cloning of *rcsD* promoter region (from position -220 relative to *rcsD* ATG) | This study |
| W4726 | CAGTTGAATTCACGGAGCGCGTGTTAAGTTG | Cloning of *rcsD* promoter region (from position -110 relative to *rcsD* ATG) | This study |
| W4798 | GCGAATTAATACGACTCACTATAGGGCTTAAGTATAAGGAGGAAAAAATATGCTTATGCGCGATGTCACCGGGCCTATCCG | Generation of *barA_198-918_-HA* template | This study |
| W4807 | CAGTTGAATTCCTACCTGCCTAAAACTATCAC | Cloning of *rcsD* promoter region (from position -270 relative to *rcsD* ATG) | This study |
| W4808 | CAGTTGAATTCCTCCCCTGCTCGACCGTTCGTAAGACATTAGCAAATAATTTCTTG | Cloning of *rcsD* promoter region (from position -235 relative to *rcsD* ATG) | This study |
| W4817 | CAGTTGAATTCCACTATTTTAGTTGCGAATGAAG | Cloning of *ompC* promoter region | This study |
| W4818 | GCATCGGATCCCAGTCGGCAAGTCCATTCTCCGCA | Cloning of *ompC* promoter region | This study |
| W4954 | CAGTTGAATTCTATAAGGAGGAAAAAATATGCTTATGCGCGATGTCACCGGGCCTATCCG | Cloning of *barA_198-918_* | This study |
